# Cognitive performance shows domain specific associations with regional cortical thickness in multiple sclerosis

**DOI:** 10.1016/j.nicl.2021.102606

**Published:** 2021-02-24

**Authors:** Jan-Patrick Stellmann, Nadine Wanke, Adil Maarouf, Susanne Gellißen, Christoph Heesen, Bertrand Audoin, Stefan M. Gold, Wafaa Zaaraoui, Jana Poettgen

**Affiliations:** a Institut für Neuroimmunologie und Multiple Sklerose, Universitätsklinikum Hamburg-Eppendorf, Martinistr. 52, 20246 Hamburg, Germany; b Klinik und Poliklinik für Neurologie, Universitätsklinikum Hamburg-Eppendorf, Martinistr. 52, 20246 Hamburg, Germany; c APHM, Hopital de la Timone, CEMEREM, Marseille, France; d Aix Marseille Univ, CNRS, CRMBM, Marseille, France; e Department of Cognitive Psychology, Institute of Psychology, University of Hamburg, Von-Melle-Park 5, 20146 Hamburg, Germany; f Department of Diagnostic and Interventional Neuroradiology, University Medical Center Hamburg Eppendorf, Hamburg, Martinistr. 52, 20246 Hamburg, Germany; g Charité Universitätsmedizin Berlin, Klinik für Psychiatrie und Psychotherapie, Campus Benjamin Franklin, Hindenburgdamm 30, 12203 Berlin, Germany; h Charité Universitätsmedizin Berlin, Medizinische Klinik m.S. Psychosomatik, Campus Benjamin Franklin, Hindenburgdamm 30, 12203 Berlin, Germany

**Keywords:** Multiple Sclerosis, MRI, Cognition, Cortical thickness

## Abstract

Multiple Sclerosis (MS) patients often suffer from significant cognitive impairment. Earlier research has shown relationships between regional cortical atrophy and cognitive deterioration. However, due to a large number of neuropsychological assessments and a heterogenous pattern of cognitive deficits in MS patients, reported associations patterns are also heterogenous. Using an extensive neuropsychological battery of 23 different tasks, we explored domain (attention/information processing, memory, spatial processing, executive functioning) and task-specific associations with regional cortical thickness in a representative sample of MS patients (N = 97). Cortical regions associated with multiple cognitive tasks in the left hemisphere were predominantly located in the inferior insula (attention p < 0.001, memory p = 0.047, spatial processing p = 0.004, executive functioning p = 0.037), the gyrus frontalis superior (attention p = 0.015, memory p = 0.037, spatial processing p = 0.033, executive functioning p = 0.017) and temporal medial (attention p < 0.001, memory two clusters p = 0.016 and p < 0.001, executive functioning p = 0.016). In the right hemisphere, we detected the strongest association in the sulcus interparietalis with five cluster (attention SDMT p = 0.003 and TAP_DA p < 0.001; memory Rey recall p = 0.013 and VLMT verbal learning p = 0.016; spatial processing Rey copy p < 0.001). We replicated parts of our results in an independent sample of 30 mildly disabled MS patients. Moreover, comparisons to 29 healthy controls showed that the regional associations seemed to represent rather pathophysiological dependency than a physiological one. We believe that our results may prove useful in diagnosis and rehabilitation of cognitive impairments and may serve as guidance in future magnetic resonance imaging (MRI) studies.

## Highlights

• Cognitive impairment correlates with loss of cortical thickness in MS.
• Cognitive tests show distinctive regional associations with cortical thickness.
• Some regions, such as the left insula, correlate with multiple tests.
• Associations patterns seem reproducible in patients with very mild impairment.
• Better localization of cognitive functions may improve future MRI studies.

## 1 Introduction

Multiple sclerosis (MS) is an autoimmune disease with a neurodegenerative component, which imposes a number of burdens on lives of patients, often including significant cognitive impairment (Di Filippo et al., 2018). Although cognitive deficit is a common feature in MS affecting from 40 to 70% of patients with impairment in information processing speed, visuospatial abilities, attention, verbal memory and executive functions (Amato et al, 2016 J Neorol Sci), its neuroimaging correlates are difficult to identify. Cortical atrophy has been identified as an important predictor of cognitive decline and appears to explain progressive cognitive deterioration to a greater extent than conventional lesion assessment (van Munster et al., 2015). Cortical atrophy was found to be present from early stages of the disease (De Stefano et al., 2003) and a recent study shows relationships between the progression of grey matter (GM) atrophy and disability accumulation in MS (Eshaghi et al., 2018). A study by Steenwijk and colleagues (Steenwijk et al., 2016) described patterns of atrophy that were associated with cognitive impairment in a non-random fashion, suggesting several structures involving bilateral posterior cingulate, lingual cortex, temporal pole, entorhinal cortex and superior frontal gyrus to be essential to cognitive functioning in MS. More recently, the importance of cortical atrophy for cognitive decline was underlined as a predominant feature of longstanding MS while in early MS cognitive impairment was rather associated with white matter integrity (Eijlers et al., 2018). Calabrese et al. (2010) observed that cortical atrophy was restricted to frontotemporal areas in cognitively preserved MS patients. However, few studies have explored the association of regional cortical thickness and their relationship to particular cognitive domains or single tasks. One of these few studies reported significant associations between GM volume in mainly frontal and temporal areas of the brain and information processing performance and verbal recognition memory (Nocentini et al., 2014). The majority of studies that have explored the relationship between cortical atrophy and cognitive impairment involved relatively small sample sizes and have either investigated cognitive performance by comparing groups of cognitively preserved and cognitively impaired patients based on overall cognitive performance (e.g. (Riccitelli et al., 2011)) or have only looked at particular cognitive subdomains (e.g. (Morgen et al., 2007)). Regional associations patterns are of further interest in functional imaging studies investigating neuroplasticity and adaption in MS (Enzinger et al., 2016). For example, there is an ongoing debate how to interpret increased connectivity in different brain regions as this might represent compensatory mechanisms or maladaptation (Penner and Aktas, 2017, Rocca and Filippi, 2017, Silemeket al., 2020). A better localisation of cortex regions that are important for cognition in MS might help to interpret altered connectivity or activation in task-based fMRI results.

The aim of the present study was to explore associations of regional cortical thickness in MS patients in relation to cognitive performance on an extensive battery of neuropsychological tests. We aimed to identify regions specific for single tests and those with a broader association pattern with cognitive performance. Moreover, we assumed that resulting association patterns from a single representative database with a single processing pipeline would allow to reduce the uncertainties about localisation from different studies. To encounter the replication problem (Daumeret al., 2008, Ioannidis, 2005), we aimed to validate findings in an independent cohort applying the same methods.

## 2 Methods

### 2.1 Participants

**Fig. 1:**
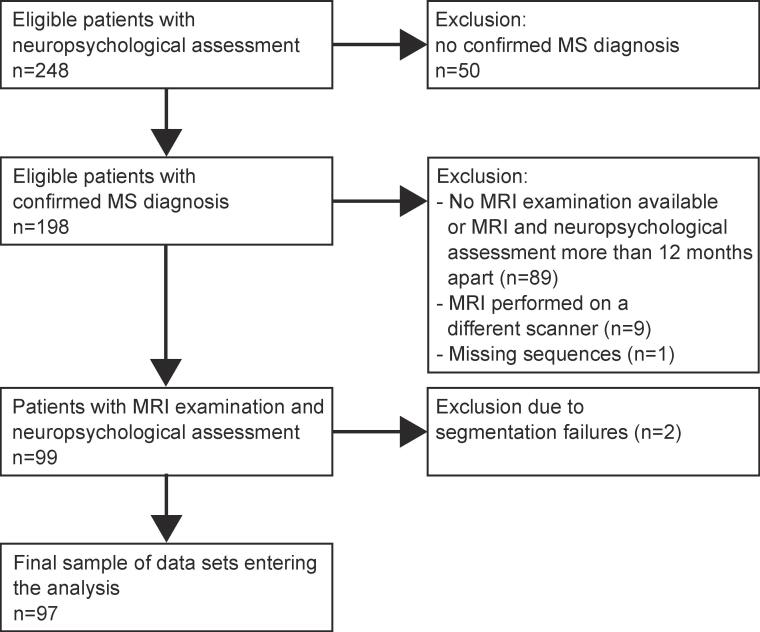
Patient selection for the explorative cohort.

The present study was a retrospective analysis of clinical and neuroimaging data available through the database of the Institute of Neuroimmunology and Multiple Sclerosis (INIMS) at the University Medical Center in Hamburg. We first identified all patients between January 2010 and December 2015 who had received a clinically indicated neuropsychological assessment at the MS outpatient clinic in Hamburg-Eppendorf at initiation of the study. Inclusion criteria were a confirmed diagnosis of MS according to the McDonald Criteria 2010 (Polman et al., 2011a) and that the MRI examination at the university clinic was not more than 12 months apart from the neuropsychological assessment (91% of the final sample included MRI examinations and neuropsychological assessments both conducted within a period of 6 months); it was further ensured that MRI data was collected using the same MRI scanner and that the respective MRI protocol included identical sequences (T1 MPRAGE, FLAIR). These criteria were met by 99 data sets, of which 2 further data sets had to be excluded due to FreeSurfer segmentation failures at a later stage, leaving a final sample of *N* = 97 for an explorative data set (Flow-chart, Fig. 1). To provide information about the specificity of our results and to validate findings in an independent dataset, we included also data from 30 relapsing-remitting MS patients and 29 healthy controls from a prospective natural history study, which underwent the same MRI assessments and the main part of the exhaustive neuropsychological assessment (details see below). The validation and healthy control cohort had been recruited between March 2013 and January 2015 at our MS outpatient clinic. Patients had to have a relapsing remitting (RR) MS in agreement with McDonald criteria 2010 (Polman et al., 2011b) without relapse or disability progression in the last 3 months. Moreover, inclusion criteria included the following: no evidence of medical illness or substance abuse that may affect cognitive functioning; no other psychiatric or neurological diseases and no change of immunotherapies within the last 3 months. The 29 healthy controls were matched based on age, sex and education. The majority of patients in the explorative cohort performed the neuropsychological testing due to subjective neuropsychological impairment or recommendation of such an examination by the treating neurologist. Thus, we assumed that the cohort had a worse cognitive performance than the validation cohort that was recruited independent of the patient’s or neurologist’s subjective impression of cognitive impairment. We further assumed that healthy controls show no impairment and perform better than the two patient cohorts. All subjects underwent neuropsychological assessment and a MRI examination within one week. All participants gave written informed consent, and the local ethic committee approved the study (Hamburg chamber of physicians, PV4405 and PV4356).

### 2.2 Neuropsychological assessment

A comprehensive battery of neuropsychological tests was used to evaluate cognitive performance in the domains attention/information processing, memory, spatial processing and executive functioning. Assessments were only performed in a stable phase of disease avoiding relapse or steroid impact on the performance. The assessment was optimized for clinical use and research (Pöttgen et al., 2013, Silemeket al., 2020; Stellmann et al., 2015; Stellmann et al., 2017). Selected tests cover in depth cognitive functions typically impaired in MS (e.g. processing speed / attention) but also other domains allowing a differential diagnostic in the clinical setting: The tests are as follows (the abbreviation VAL indicates availability of test data in all three cohorts - exploration, validation and healthy controls):

We employed three subtests of the *Test battery of Attentional Performance* (TAP; (Zimmermann and Fimm, 2002)) to evaluate attentional capacities: The alertness task (tonic and phasic alertness, VAL), the selective attention task (Go/NoGo), and the divided attention task (dual task). The *Trial Making Test A* (TMT-A; (Reitan and Wolfson, 1992, Tombaugh, 2004)) was used to evaluate information processing speed. Information processing was assessed using the oral version of the *Symbol Digit Modalities Test* (SDMT; (Smith, 1995), VAL). We employed the *Paced Auditory Serial Addition Test* (PASAT 3″; (Gronwall, 1977), VAL) to evaluate divided attention with components of working memory and numeracy skills. The *Wechsler Memory Scale (WMS-R) digits forward /backward task* (Haerting et al., 2000) was used to assess short-term memory (digits forward) and working memory (digits backward). The assessment further included the *WMS verbal logical memory* (immediate and delayed recall). The *verbal learning and memory task (VLMT*; (Helmstaedter et al., 2001), VAL) was employed to assess supraspan, verbal learning, verbal memory, and recognition of verbal material. Spatial constructive abilities were evaluated using the *Rey-Osterrieth complex figure test* (copy), and the recall condition was used to assess spatial memory (30 min delay) (Bennett-Levy, 1984). *Subtests of the “Leistungsprüfungssystem”* (LPS; (Horn, 1983)) were used to evaluate logical reasoning (LPS 3) and spatial cognitive abilities (LPS 7), respectively. The *Trial Making Test B* (TMT-B; (Reitan and Wolfson, 1992, Tombaugh, 2004)) was used to evaluate task switching. The subtest “animals” of the *Regensburger Word Fluency Task* (RWT; (Aschenbrenner et al., 2000)) with a test duration of 2 min was employed to assess word fluency (VAL), the subtest “G-R” to assess verbal cognitive flexibility. Action planning skills were evaluated using the *Zoo Task from the Behavioural Assessment of the Dysexecutive Syndrome* (BADS; (Ufer and Wilson, 2000)). For descriptive statistics of the cognitive profile of the cohorts, performance was classified as below average if test scores fell more than one standard deviation below the mean of the normative sample for the respective neuropsychological test. The classification was only used for the descriptive comparison of the cohorts and was neither used for further analyses addressing the association with cortical thickness nor as definition of cognitive impairment. All neuropsychological assessments were performed in a stable phase of the disease at least 8 weeks apart from the last relapse.

### 2.3 Self-report measures

Self-report measures were administered during the neuropsychological assessment to examine fatigue (Fatigue Scale for Motor and Cognitive Functions, FSMC; Penner and Aktas, 2017) and anxiety and depression (Hospital Anxiety and Depression Scale, HADS; (Honarmand and Feinstein, 2009)).

### 2.4 MRI protocol

For all cohorts, MRI data were acquired with the same 3 T MRI scanner (Skyra, Siemens Medical Systems, Erlangen, Germany). The MRI protocol included the following sequences used in all cohorts: a 3D magnetization prepared rapid acquisition gradient-echo (MPRAGE) T1 weighted sequence (TR/TE = 2500 ms/2.12 ms; TI = 1100 ms; 256 slices, voxel size 0.8 × 0.8 × 0.9 mm, matrix = 288x288, FOV = 240 mm) and a T2 FLAIR sequence (TR/TE = 9000 ms/90 ms; 43 slices, voxel size 0.7 × 0.7 × 3.0 mm, matrix = 320x320, FOV = 230 mm). All MRI were scheduled at least 4 weeks after the last steroid treatment.

### 2.5 Image analysis

Images were processed with the functional imaging software library (FSL, version 5.0, www.fmrib.ox.ac.uk). Images were reoriented to standard MNI space and T1 and FLAIR images were registered for lesion mapping and filling. We applied first the lesion growth algorithm (Schmidt et al., 2012) as implemented in the LST toolbox version 2.0.1 (www.statistical- modelling.de/lst.html) for SPM on FLAIR images and extracted total lesion volume. To minimize segmentation errors, we performed lesion filling on T1 weighted images by filling marked lesion areas with mean intensity values from the lesions’ surrounding parenchyma. Lesion-filled T1-weighted images were automatically processed with FreeSurfer software (Version 5.2.0) for cortical reconstruction and volumetric segmentation (surfer.nmr.mgh.harvard.edu/). To minimize segmentation errors, one individual supervised by JPS manually corrected brain masks and white / grey matter segmentation in all cases. However, two data sets had to be excluded from the analysis due to persisting segmentation failures (see Fig. 1). Spatial normalization was performed vertex-wise and a cortical thickness map was created for each subject. The individual maps were registered to the FreeSurfer ‘fsaverage’ template and smoothed with a Gaussian kernel of 10 mm full-width at half maximum (FWHM).

### 2.6 Statistics

Besides descriptive statistics of the cohorts we used Student’s T-test and Fischer’s exact tests to compare them. Moreover, we used the permutation version (1000 permutations) of the Jonckheere-Terpstra test for ordered differences among groups to proof difference in cognitive performance between the three cohorts: We hypothesized that healthy controls have the best cognitive performance followed by the validation cohort and finally the explorative cohort with the highest impairment. The FreeSurfer application QDEC (Query, Design, Estimate, Contrast; www.surfer.nmr.mgh.harvard.edu) was employed to perform inter-subject averaging and inference on cortical thickness. A General Linear Model (GLM) was used to correlate cortical thickness with raw scores on neuropsychological tasks while controlling for sex, education level and age (analysis I) and sex, education level, age and lesion volume (analysis II). The second analysis was included to decipher the impact of neurodegeneration alone i.e., how much cortical thickness loss is independent from the general inflammatory activity. We included right-handed performance on the 9-hole-peg-test (9-HPT) as an additional covariate for tests relying on motor function (TMT-A, TMT-B, and TAP subtests). Monte Carlo simulations were applied to clusterwise correct for multiple comparisons (Hagler et al., 2006). The procedure involved 10 000 repetitions and a threshold of p = 0.001 (one-sided testing). Reported standardized Beta-values for each cluster were corrected such as a positive value always indicates better cognitive performance is associated with higher thickness. Clusterwise p-values from the GLM are reported as well. Moreover, we extracted the location of the clusters based on the Desikan-Killiany Atlas. Resulting maps for each test were combined by cognitive domains and as a total map. GLM was further used to identify regions with significant loss of cortical thickness in MS patients compared to controls. All resulting association maps (single tasks, domains and summary map) are freely available for download as FreeSurfer annotation and volume files (github.com/oneeq/ms_cognition_atlas). The data that support the findings of this study are available from the corresponding author upon reasonable request.

For the validation and control cohorts we applied the same imaging processing pipeline with FSL and FreeSurfer. Afterwards we applied the atlas from the explorative cohort on control and validation images to extracted cortical thickness values. We included all clusters with significant results in the explorative cohort and task results in the validation and control cohort (four tests). We used statistics in R to compute Pearson’s correlations between cortical thickness and tasks scores (corrected for age, sex and education level) to confirm associations between cognitive performance and cortical thickness in every single cluster. We considered p-values below 0.05 as significant after applying the false discovery rate (FDR) correction.

## 3 Results

### 3.1 Demographic data

Demographic and clinical data are displayed in table 1. The validation cohort showed a tendency towards higher education (p = 0.05) but did not differ from the explorative cohort concerning age and sex. In contrast to the explorative cohort, patients in the validation cohort had a lower disability level (p = 0.001), while disease duration, lesion numbers and lesion volumes were comparable between the groups. The healthy controls group was matched by sex, age and education to the validation cohort and did not differ from the explorative cohort for these baseline characteristics.

**Table 1:** Demographic and clinical Data.

	Explorative cohort N = 97	Validation cohort N = 30	Controls N = 29	E vs V	V vs C	E vs C
**Age***years*	39.2 (10.6)	40.3 (9.9)	40.1 (8.7)	0.603^§^	0.947^§^	0.632^§^
**Female** n(%)	60 (61.9%)	21 (70%)	18 (62%)	0.622^§^	0.713^§^	1.0^§^
**Years of education***12 or more* n(%)	47 (48.5%)	21 (70%)	17 (58.6%)	0.050^§^	0.522^§^	0.397^§^
**Disease duration***years*	7.4 (6.9)	9.3 (8.3)		0.278^§^		
**Disease course** n(%) relapse remitting primary progressive secondary progressive not specified	79 (81.4%) 8 (8.2%) 7 (7.2%) 3 (3.1%)	30 (100%)		0.004^#^		
**EDSS** median [range]	2.5 [0–7]	2 [0–4]		0.001^§^		
**T2-Lesion volume***ml*	6.0 (9.2)	9.9 (12.4)		0.155^§^		
**Number of lesions**	32.2 (20)	35.8 (19.0)		0.410^§^		
**Time difference MRI - neuropsychology***days*	76.9 (81.9)					
**MRI and neuropsychology within 6 months** n (%)	88 (92%)					

Demographic data as mean (SD) if not otherwise indicated, EDSS = Expanded Disability Status Scale, disease duration since first symptoms. Task scores represent raw scores. No missing tests for the validation cohort and controls, thus n = 30 respectively n = 29 for all available tests. ms = milliseconds, group comparison with Student’s T-test^§^ or Fisher’s exact^#^.

### 3.2 Cognitive profile of the cohorts

A comparison of the cognitive profiles of the explorative cohort and the validation cohort revealed a rather homogeneous pattern such as the explorative cohort was overall more cognitively disabled and performed worse than the validation cohort. However, the validation cohort still scored below controls (Jonckheere-Terpstra test for an ordered differences among the three groups for all but one test: p < 0.005)). Patients from the explorative cohort were most severely impaired on attention tasks, with almost 60% of patients (n = 56, TAP_DA) presenting scores in divided attention task below mean (table 2). Moreover, half of our sample showed low performance in selective attention (n = 46, TAP_TA) and phasic alertness tasks (n = 52, TAP_PA), whereas a slightly smaller proportion of patients showed such scores on the tonic alertness task (n = 42, TAP_TA). A substantial number of patients performed more than 1 SD below mean on the SDMT (41.7%, n = 40), and less than one third of the sample showed task results more than 1 SD below mean on the TMT-A (n = 30) and PASAT (n = 21). Likewise, large proportions of patients were performing similar on executive functioning. More than half of our sample showed reduces performance in tasks measuring cognitive flexibility and task switching (RWT-GR; n = 53 and TMT-B; n = 49), and more than one third scored below average on word fluency (RWT-animals, n = 36) and planning skills (Zoo task, n = 36). In contrast, only very few patients presented low scores in a logical reasoning task (LPS3, n = 2). In the memory domain, proportions of performance more than 1 SD below the mean ranged from about 10% − 30%, with fewest patients on figural memory (Rey delay, n = 9) and most patients in short-term memory (WMS Digits FW, n = 27) and working memory (WMS Digits BW, n = 29). Finally, <10% of our sample showed low tasks results in spatial processing (LP7; n = 7 and Rey copy; n = 9).

**Table 2:** Neuropsychological Data.

		Explorative cohort				Validation cohort			Controls			E < V < C
Task	Cognitive function	N	Median [Range]	Mean (SD)	below mean n (%)	Median [Range]	Mean (SD)	below mean n (%)	Median Range	Mean SD	below mean n (%)	
**Attention/information processing**												
TAP_PA (*ms)*	Phasic alertness	97	265 [198; 1001]	290 (99)	52 (53.6%)	249 [185; 340]	256 (41)	13 (43.3%)	238 [202; 299]	244 (26)	6 (20.7%)	0.001
TAP_TA (*ms)*	Tonic alertness	97	267 [202; 964]	290 (99)	42 (43.3%)	253 [196; 368]	261 (43)	8 (26.7%)	241 [198; 330]	246 (30)	3 (10.3%)	0.002
TAP_SA (*ms)*	Selective attention	94	461 [275; 886]	467 (1 0 4)	46 (49.5%)							
TAP_DA (*ms)*	Divided attention	94	730 [508; 1181]	738 (1 2 2)	56 (59.6%)							
PASAT	Selective attention (and working memory / calculating capacity)	74	47 [0; 60]	44.4 (11.6)	21 (28%)	53[30; 60]	48.7 (10.4)	8 (26.7%)	54 [41; 60]	52.4 (6.1)	0 (0%)	0.001
SDMT	Information processing	93	55 [17; 85]	53.8 (13.4)	40 (41.7%)	55 [32; 108]	58.1 (14.7)	6 (20%)	60 [45; 94]	63.0 (12.4%)	0 (0%)	0.003
TMT-A	Information processing speed	97	30.6 [13.3; 146]	34.4 (17.3)	30 (30.9%)							
**Memory**												
Rey delay	Figural memory	93	20.5 [5.5; 36]	20.0 (6.2)	9 (9.3%)							
VLMT 1	supraspan	90	6.5 [1; 12]	6.5 (2.1)	14 (15.6%)	7.0 [4; 15]	7.6 (2.5)	2 (6.7%)	9.0 [6; 15]	9.0 (2.1)	0 (0%)	0.001
VLMT 1–5	Verbal learning	97	53 [5.5; 69]	50.4 (11.3)	25 (25.8%)	53 [35; 75]	54.1 (9.7)	4 (13.3%)	62 [50; 75]	63.0 (6.3)	0 (0%)	0.001
VLMT_5-7	Verbal memory	97	1 [-2; 9]	1.2 (2.0)	15 (15.5%)	1.5 [-3; 5]	1.1 (2.0)	4 (13.3%)	0.0 [-2; 5]	0.5 (1.5)	1 (3.4%)	0.879
VLMT recognition	Recognition of learned material	96	14 [(-1); 15]	12.5 (3.2)	12 (12.5%)							
WMS (delayed recall)	Verbal logical memory (delayed recall)	94	29 [6; 45]	27.6 (7.9)	16 (17.0%)							
WMS (immediate recall)	Verbal logical memory (immediate recall)	95	32 [7; 45]	30.4 (7.5)	15 (15.8%)							
WMS Digits BW	Working memory	97	6 [2; 10]	6.1 (1.8)	29 (29.9%)							
WMS Digits FW	Short-term memory	97	7 [3; 11]	7.2 (1.7)	27 (27.8%)							
**Spatial processing**												
LPS 7	Spatial-cognitive abilities	96	17.5 [5; 34]	18.4 (6.8)	7 (7.3%)							
Rey copy	Spatial-constructive abilities	93	35 [23; 36]	34.1 (2.9)	9 (9.7%)							
**Executive functioning**												
LPS 3	Logical reasoning	96	25.5 [7; 39]	25.1 (5.4)	2 (2.1%)							
RWT-GR	Cognitive flexibility	97	17 [4; 35]	17.6 (6.1)	53 (54.6%)							
RWT-animals	Word fluency	97	34 [10; 59]	34 (11.0)	36 (37.1%)	36 [17; 64]	36 (11.0)	7 (23.3%)	41 [25; 72]	42.1 (9.6)	1 (3.4%)	0.001
TMT-B	Task switching	96	71.6 [31; 278]	82.6 (44.8)	49 (51.0%)							
BADS Zoo Task	Planning skills	91	3 [0; 214]	5.1 (22.2)	36 (39.6%)							

Cognitive profiles of the three cohorts. N indicates the number of patients with data in the explorative cohort. Scoring below mean defined by scoring below −1 SD. The expected value from a normal distribution is that only 16% score equals or below −1SD. E < V < C reports p-values from the Jonckheere-Terpstra test for the hypothesis that the cognitive performance in the explorative cohort is worth than in the validation cohort, while healthy controls perform best.

### 3.3 Association between cognitive tests and cortical thickness in the explorative cohort

**Fig. 2:**
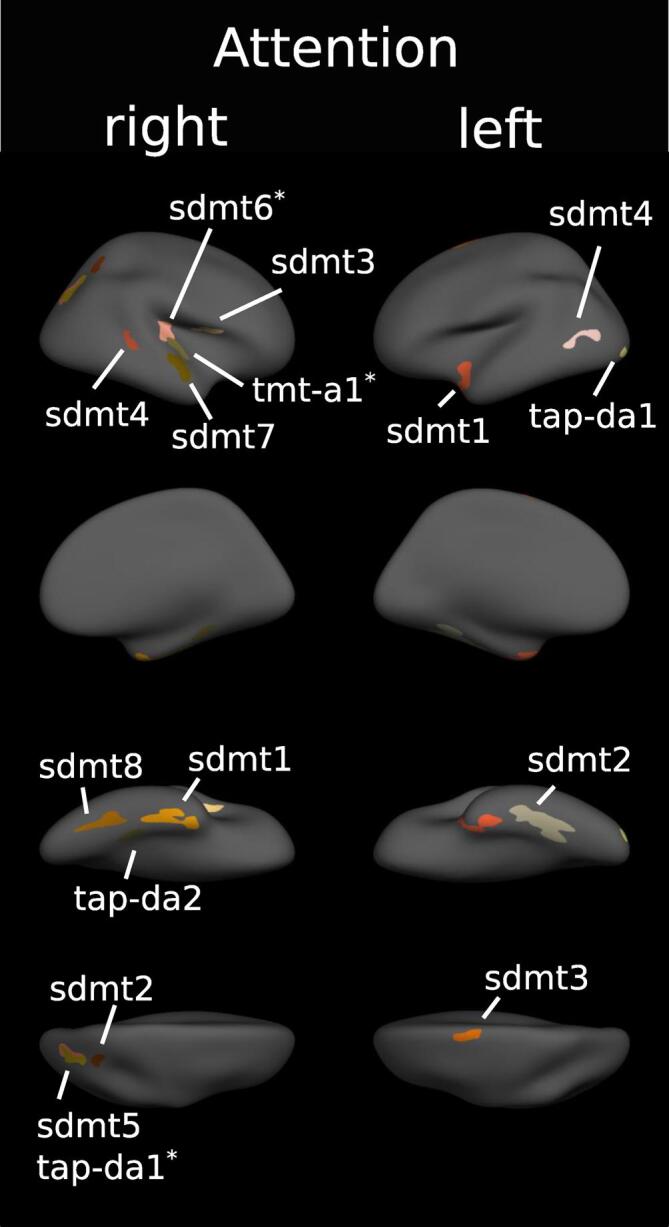
Cortical regions associated with attention. Clusters of association between cortical thickness and cognitive tests of attention displayed on the FreeSurfer fsaverage surface. Numbers correspond to cluster index in table 4. * = also significant if corrected for T2 lesions. For separated results of each test see appendix.

**Fig. 3:**
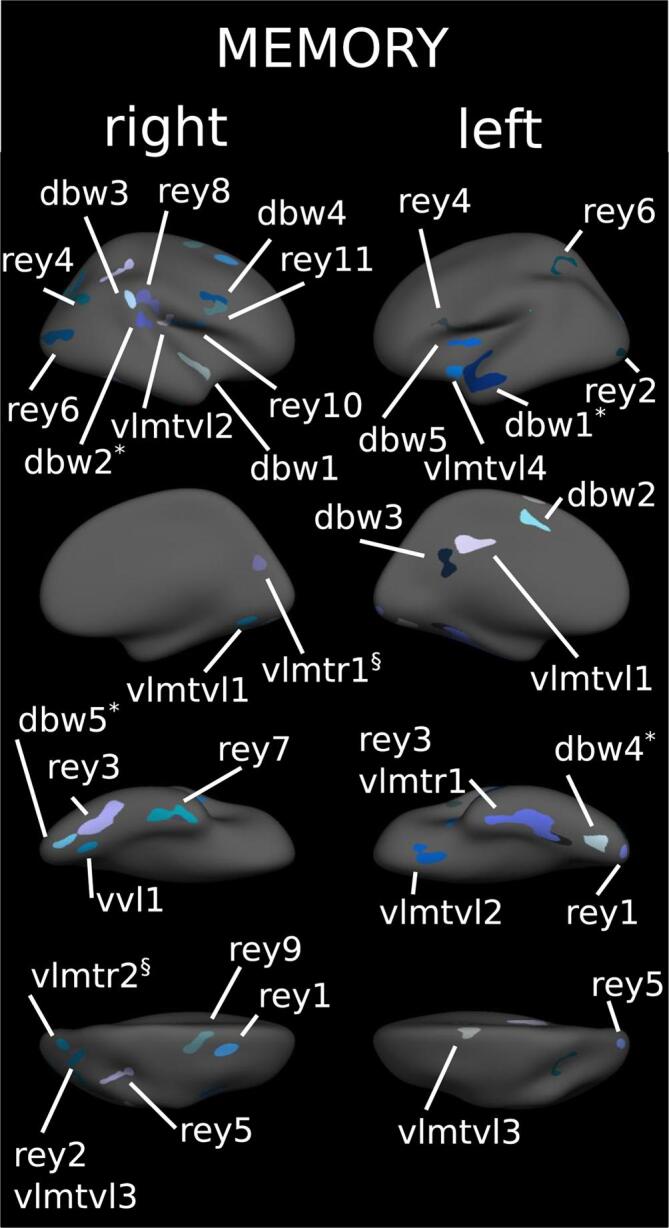
Cortical regions associated with memory. Clusters of association between cortical thickness and cognitive tests of memory displayed on the FreeSurfer fsaverage surface. Numbers correspond to cluster index in table 5. * = also significant if corrected for T2 lesions, § = only significant if corrected for T2 lesions. For separated results of each test see appendix.

**Fig. 4:**
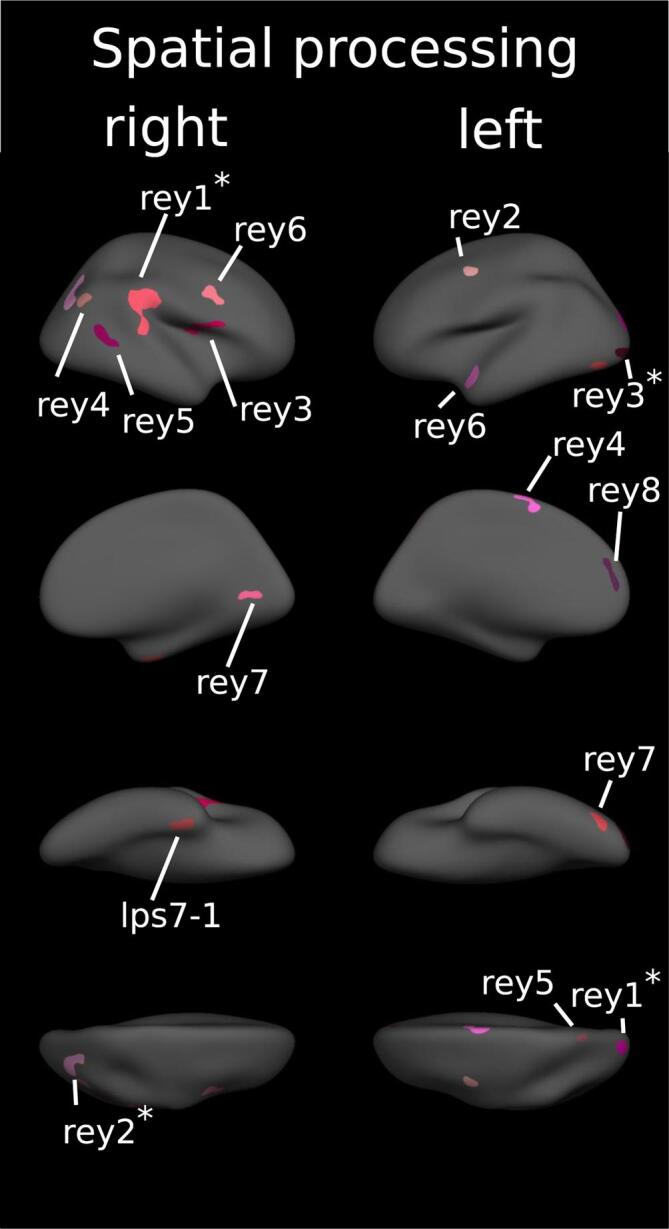
Cortical regions associated with spatial processing. Clusters of association between cortical thickness and cognitive tests of spatial processing displayed on the FreeSurfer fsaverage surface. Numbers correspond to cluster index in table 6. * = also significant if corrected for T2 lesions. For separated results of each test see appendix.

**Fig. 5:**
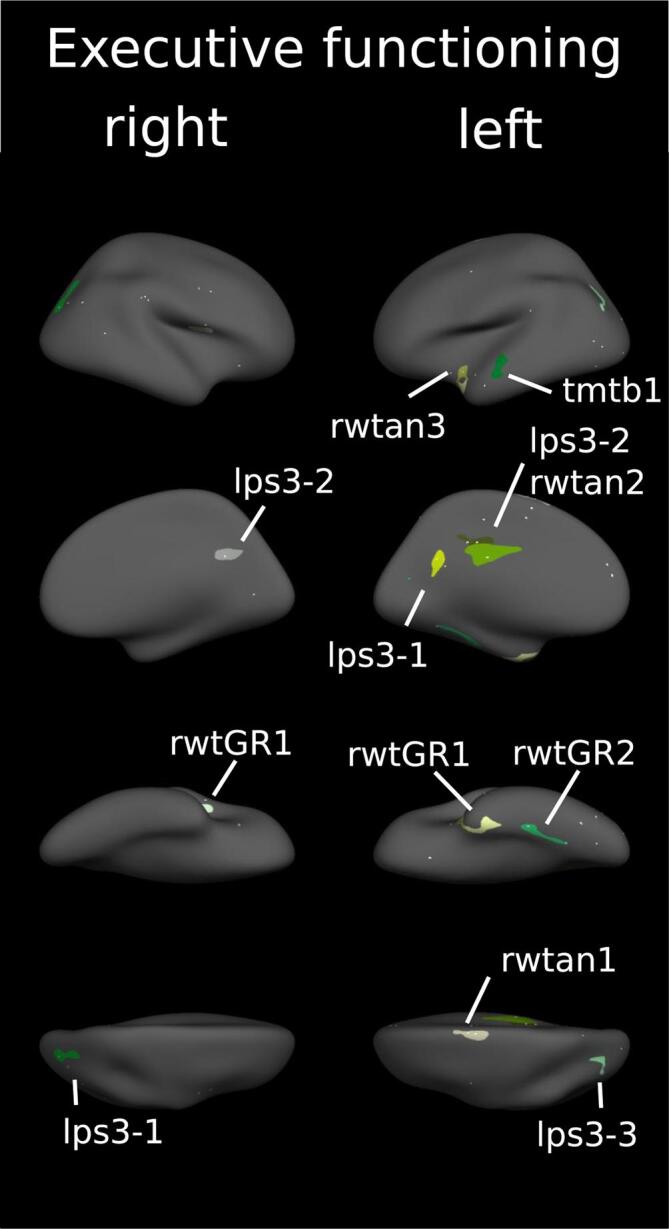
Cortical regions associated with executive functioning. Clusters of association between cortical thickness and cognitive tests of executive functioning displayed on the FreeSurfer fsaverage surface. Numbers correspond to cluster index in table 5. For separated results of each test see appendix.

We investigated the correlation between cortical thickness and single task scores to identify cortical clusters of associations. Results are summarized along four cognitive domains, each with a representative figure showing all the clusters of a domain. Statistics including beta and values are summarised in a corresponding table: For the domain of attention / information processing in Fig. 2 and Table 3, for memory in Fig. 3 and Table 4, for spatial processing in Fig. 4 and Table 5 and executive functioning in Fig. 5 and Table 6. More details including figures of significant clusters for each task are available within the appendix. There, we provide also a brief review of our task specific findings with regards to the available literature. All findings are also available as label and annotations files in a GitHub repository (github.com/oneeq/ms_cognition_atlas).

**Table 3:** Cortical regions associated with attention/information processing.

		**Analysis I**				**Analysis II**		
**Task**	**Hemisphere**	**Region**	**Cluster index**	**p**	**Beta (SD)**	**Region**	**P**	**Beta (SD)**
**TAP_PA**	LH	NO CLUSTERS						
	RH	NO CLUSTERS						
**TAP_TA**	LH	NO CLUSTERS						
	RH	NO CLUSTERS						
**TAP_SA**	LH	NO CLUSTERS						
	RH	NO CLUSTERS						
**TAP_DA**	LH	lateraloccipital	1	0.027	3.001 (0.154)			
	RH	Superiorparietal	1	<0.001	2.745 (0.191)	Superiorparietal	0.012	3.138 (0.093)
		Parahippocampal	2	0.028	2.966 (0.148)	Lateraloccipital	0.004	3.177 (0.232)
**SDMT**	LH	Fusiform	1	<0.001	2.556 (0.667)			
		Fusiform	2	<0.001	2.055 (0.324)			
		Superiorfrontal	3	0.014	1.928 (0.107)			
		Lateraloccipital	4	0.049	2.072 (0.196)			
	RH	Fusiform	1	<0.001	2.475 (0.521)			
		Superiorparietal	2	0.034	1.909 (0.207)			
		Parsopercularis	3	0.011	2.176 (0.228)			
		Bankssts	4	0.045	2.231 (0.267)			
		Inferiorparietal	5	0.003	1.665 (0.094)			
		Insula	6	0.001	1.983 (0.292)			
		Superiortemporal	7	<0.001	2.550 (0.222)			
		Fusiform	8	0.002	1.620 (0.278)			
						Transversetemporal	0.011	0.001 (0.001)
**TMT-A**	LH	NO CLUSTERS						
	RH	Transversetemporal	1	0.009	2.538 (0.231)			
**PASAT**	LH	NO CLUSTERS						
	RH	NO CLUSTERS						

Summary of cortical clusters with a significant correlation between cortical thickness and task scores from the domain of attention/information processing adjusted for age, sex and education (Analysis I). Analysis II includes an additional adjustment for T2 lesions. Beta-values for each cluster were corrected such as a positive value always indicates better task performance is associated with higher thickness. p-values after correcting for multiple testing. Regions correspond to the Desikan-Killiany Atlas.

**Table 4:** Cortical regions associated with memory.

		**Analysis I**				**Analysis II**		
**Task**	**Hemisphere**	**Region**	**Cluster index**	**p**	**Beta (SD)**	**Region**	**P**	**Beta (SD)**
**WMS Digit FW**	LH	NO CLUSTERS						
	RH	NO CLUSTERS						
**WMS Digit BW**	LH	Middletemporal*	1	<0.001	2.338 (0.350)	Middletemporal	<0.001	2.596 (0.452)
		Superiorfrontal	2	0.044	1.949 (0.220)			
		Isthmuscingulate	3	0.015	1.853 (0.321)			
		Lateraloccipital*	4	0.003	1.493 (0.239)	Lateraloccipital	<0.001	1.210 (0.219)
		Insula	5	0.050	2.460 (0.223)			
	RH	Superiortemporal	1	0.002	2.529 (0.116)			
		Superiortemporal*	2	0.007	2.044 (0.180)	Superiortemporal	0.037	1.919 (0.229)
		Supramarginal	3	0.035	2.134 (0.320)			
		Precentral	4	0.021	2.258 (0.113)			
		Lateraloccipital*	5	0.035	1.623 (0.228)	Lateraloccipital	0.027	1.585 (0.126)
**WMS VLM immediate**	LH	NO CLUSTERS						
	RH	NO CLUSTERS						
**WMS VLM delayed**	LH	Fusiform	1	0.016	1.955 (0.242)			
	RH	Fusiform	1	0.022	2.910 (0.121)			
**Rey recall**	LH	Lateraloccipital	1	0.046	1.451 (0.257)			
		Lateraloccipital	2	0.027	2.095 (0.114)			
		Fusiform	3	<0.001	1.911 (0.357)			
		Parsopercularis	4	0.017	2.283 (0.167)			
		Superiorparietal	5	0.045	1.651 (0.181)			
		Superiorparietal	6	0.032	1.871 (0.145)			
	RH	Superiorfrontal	1	0.030	1.639 (0.199)			
		Superiorparietal	2	0.013	1.832 (0.164)			
		Fusiform	3	<0.001	1.885 (0.326)			
		Inferiorparietal	4	0.010	2.084 (0.323)			
		Superiorparietal	5	0.004	2.073 (0.281)			
		Lateraloccipital	6	<0.001	1.689 (0.455)			
		Inferiortemporal	7	<0.001	2.152 (0.455)			
		Supramarginal	8	0.002	2.533 (0.229)			
		Precentral	9	0.006	1.940 (0.507)			
		Precentral	10	0.018	2.273 (0.198)			
		parsopercularis	11	0.037	2.407 (0.339)			
**VLMT supraspan**	LH	NO CLUSTERS						
	RH	NO CLUSTERS						
**VLMT verbal learning**	LH	isthmuscingulate	1	0.004	0.476 (0.835)			
		lateralorbitofrontal	2	0.017	1.819 (0.170)			
		superiorfrontal	3	0.037	1.560 (0.204)			
		insula	4	0.047	2.870 (0.560)			
	RH	lingual	1	0.030				
		supramarginal	2	0.035				
		inferiorparietal	3	0.016				
**VLMT verbal memory**	LH	NO CLUSTERS						
	RH	NO CLUSTERS						
**VLMT recognition**	LH	NO CLUSTERS						
	RH					Cuneus	0.020	1.866 (0.147)
						superiorparietal	0.008	2.139 (0.321)

Summary of cortical clusters with a significant correlation between cortical thickness and task scores from the domain of memory adjusted for age, sex and education (Analysis I). Analysis II includes an additional adjustment for T2 lesions. Beta-values for each cluster were corrected such as positive values always indicate better task performance = higher thickness. p-values after correcting for multiple testing. Regions defined according to the Desikan-Killiany Atlas.

**Table 5:** Cortical regions associated with spatial processing.

		**Analysis I**				**Analysis II**		
**Task**	**Hemisphere**	**Region**	**Cluster index**	**p**	**Beta (SD)**	**Region**	**P**	**Beta (SD)**
**LPS 7**	LH	NO CLUSTERS						
	RH	fusiform	1	0.003	2.591 (0.389)			
**Rey copy**	LH	Lateraloccipital	1	0.002	1.454 (0.250)	Lateraloccipital	0.012	1.617 (0.151)
		Precentral	2	0.049	3.110 (0.157)			
		Lateraloccipital	3	<0.001	2.088 (0.347)	Lateraloccipital	0.002	2.401 (0.379)
		Superiorfrontal	4	0.033	2.952 (0.552)			
		Superiorparietal	5	0.037	1.950 (0.300)			
		Superiortemporal	6	0.004	2.392 (0.357)			
		Lateraloccipital	7	0.011	3.111 (0.717)			
		Superiorfrontal	8	0.030	2.918 (0.125)			
	RH	Supramarginal	1	<0.001	2.343 (0.298)	Supramarginal	<0.001	2.262 (0.366)
		Inferiorparietal	2	<0.001	1.801 (0.253)	Inferiorparietal	0.020	
		Parsopercularis	3	<0.001	2.561 (0.506)			
		Inferiorparietal	4	0.026	2.002 (0.181)			2.152 (0.237)
		Middletemporal	5	0.006	2.662 (0.274)			
		Precentral	6	0.015	2.389 (0.307)			
		pericalcarine	7	0.040	1.765 (0.320)			

Summary of cortical clusters with a significant correlation between cortical thickness and task scores from the domain of spatial processing adjusted for age, sex and education (Analysis I). Analysis II includes an additional adjustment for T2 lesions. Beta-values for each cluster were corrected such as positive values always indicate better task performance = higher thickness. p-values after correcting for multiple testing. Regions defined according to the Desikan-Killiany Atlas.

**Table 6:** Cortical regions associated with executive functioning.

		**Analysis I**				**Analysis II**		
**Task**	**Hemisphere**	**Region**	**Cluster index**	**p**	**Beta (SD)**	**Region**	**P**	**Beta (SD)**
**TMT-B**	LH	Superiortemporal	1	0.004	3.068 (0.203)			
	RH	NO CLUSTERS						
**LPS 3**	LH	Precuenus	1	0.043	2.389 (0.138)			
		Posteriorcingulate	2	0.022	0.630 (0.703)			
		Inferiorparietal	3	0.035	1.825 (0.271)	Inferiorparietal	0.028	1.309 (0.282)
	RH	Superiorparietal	1	0.004	1.474 (0.098)			
		Precuneus	2	0.037	2.461 (0.268)			
**RWT-animals**	LH	Superiorfrontal	1	0.017	2.033 (0.194)			
		Posteriorcingulate	2	0.003	0.409 (0.473)			
		Temporalpole	3	0.037	2.265 (0.209)			
	RH	NO CLUSTERS						
**RWT-GR**	LH	Fusiform	1	<0.001	3.121 (0.500)			
		Fusiform	2	0.016	2.537 (0.228)			
	RH	Precentral	1	0.014	2.641 (0.075)			
**BADS Zoo Task**	LH	NO CLUSTERS						
	RH	NO CLUSTERS						

Summary of cortical clusters with a significant correlation between cortical thickness and task scores from the domain of executive functioning adjusted for age, sex and education (Analysis I). Analysis II includes an additional adjustment for T2 lesions. Beta-values for each cluster were corrected such as positive values always indicate better task performance = higher thickness. p-values after correcting for multiple testing. Regions defined according to the Desikan-Killiany Atlas.

### 3.4 Left insula and right sulcus interparietalis are associated with multiple tasks in the explorative cohort

**Fig. 6:**
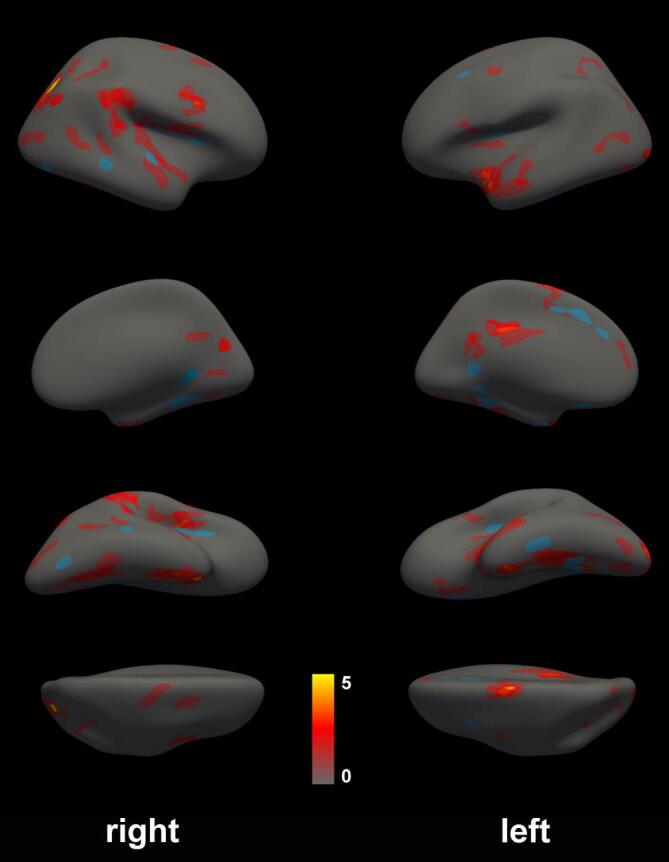
All cortical regions associated with cognitive functioning in the explorative cohort and cortical thickness in comparison to healthy controls. Overlay of all clusters displayed on the FreeSurfer fsaverage surface. The red to yellow color scale indicates the number of overlaying clusters. Regions with significant lower cortical thickness in the validation cohort in comparison to healthy controls are visualized in blue. (For interpretation of the references to color in this figure legend, the reader is referred to the web version of this article.)

We summarized our findings by merging all significant clusters in one map (Fig. 6). Cortical regions associated with multiple cognitive tasks in the left hemisphere were predominantly located in the inferior insula (attention: SDMT cluster 1p < 0.001, memory: VLMT verbal learning cluster 4p = 0.047, spatial processing: Rey copy cluster 6p = 0.004, executive functioning: RWT animals cluster 3p = 0.037), the gyrus frontalis superior (attention: SDMT cluster 3p = 0.015, memory: VLMT verbal learning cluster 3p = 0.037, spatial processing: Rey copy cluster 4p = 0.033, executive functioning: RWT animals cluster 1p = 0.017) and temporal medial (attention: SDMT cluster 2p < 0.001, memory: VLM delayed cluster 1p = 0.016 and Rey recall cluster 3p < 0.001, executive functioning: RWT GR cluster 2p = 0.016). Further regions with at least three overlaying clusters were located at the occipital pole, the gyrus cingulum, the sulcus occipo-temporal and gyrus frontalis superior.

In the right hemisphere, we detected the strongest association in the sulcus interparietalis where five clusters showed an overlay: For the domain of attention SDMT cluster 5p = 0.003 and TAP_DA cluster 1p < 0.001; for memory Rey recall cluster 2p = 0.013 and VLMT verbal learning cluster 3p = 0.016; for spatial processing Rey copy cluster 2p < 0.001). An overlap of at least three clusters could be identified for the inferior precentral sulcus, the insula and the sulcus collateralis transversum,

### 3.5 Minor overlap of atrophic brain regions and clusters associated with cognitive tasks

*Next, we investigated if the clusters identified in the explorative cohort show an overlap with loss of cortical thickness in comparison to healthy controls. We observed in total 16 regions with a significant cortical thickness loss in MS (*Fig. 6*): 10 regions in the left hemisphere (p values between < 0.001 and 0.031) and 6 regions in the right hemisphere (p values between < 0.001 and 0.031). For both hemispheres the largest region with cortical loss was located temporomedial (p < 0.001). Four out of six regions with cortical thickness loss in the right hemisphere had an overlap with at least one cognitive cluster: lateral superior temporal gyrus, circular sulcus of insula, posterior part of the lateral fissure and lingual gyrus. In the left hemisphere, 4 from 10 regions with reduced cortical thickness showed an overlap with task specific regions. Overall, the overlap was rather small as illustrated in* Fig. 6*.*

### 3.6 Lack of association in healthy controls indicates MS specificity of findings from explorative cohort

**Fig. 7:**
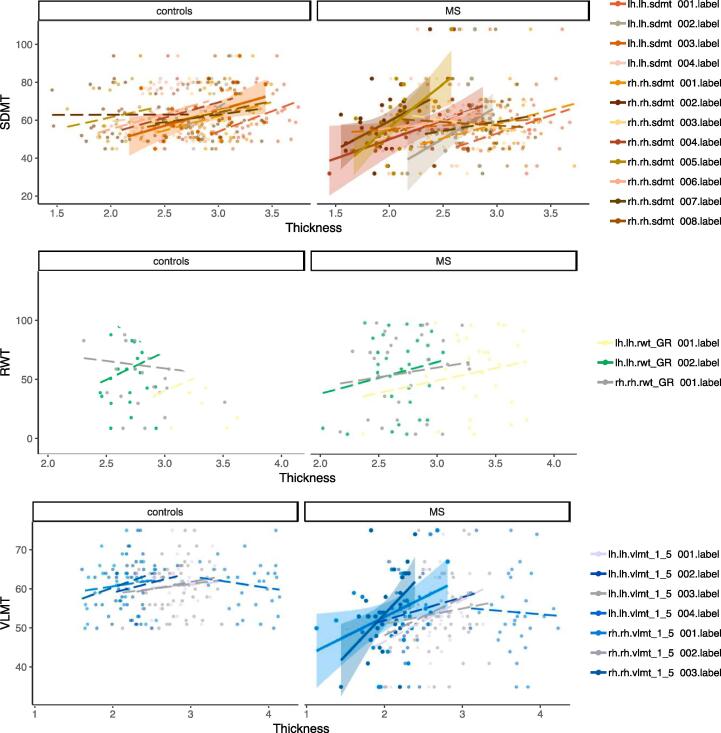
Reproducibility and application of the atlas in relapsing remitting MS and controls. Correlation between extracted cortical thickness based on the identified regions and cognitive performance in controls (left) and relapsing remitting MS patients (right). Continuous regression lines and shaded confidence intervals indicate significance. Dashed lines – not significant.

Three cognitive tests were available for validation i.e. had significant clusters and were available in healty subjects and the validation cohort: For the cognitive domain of attention/information processing we could analyse the SDMT, executive functioning was represented by RWT “animals” while the VLMT verbal learning task could be analysed for memory (Fig. 7 and table 7, for details see appendix). For healthy controls, we found only one significant association between SDMT performance and cortical thickness extracted from SDMT cluster No. 3 in the left superiorfrontal gyrus (p = 0.020). For all other clusters from the explorative cohort we found no correlation between test results and cortical thickness in healthy controls.

**Table 7:** Cluster validation: Cortical thickness and cognitive functioning in healthy controls and the validation cohort.

			**Healthy controls**		**Validation cohort**		**ANOVA**
**Task**	**Hemisphere**	**Cluster index**	**r**	**p**	**r**	**p**	
**SDMT**	RH	1	0.28	0.148	0.27	0.145	0.994
		2	0.01	0.984	0.40	0.026*	0.052
		3	0.20	0.289	−0.16	0.397	0.184
		4	0.30	0.119	0.39	0.032*	0.766
		5	0.20	0.308	0.58	<0.001*^§^	0.057
		6	0.33	0.076	0.14	0.465	0.561
		7	0.14	0.460	0.17	0.364	0.915
		8	0.24	0.200	−0.06	0.741	0.285
	LH	1	0.34	0.068	0.31	0.093	0.871
		2	0.36	0.053	0.42	0.022*	0.986
		3	0.43	0.020*	0.10	0.602	0.224
		4	0.16	0.405	0.30	0.107	0.470
**RWT-animals**	LH	1	0.20	0.301	0.47	0.009*	0.572
		2	0.20	0.309	0.19	0.320	0.480
		3	0.09	0.670	−0.19	0.313	0.363
**VLMTverbal learning**	RH	1	0.14	0.484	0.40	0.030*	0.292
		2	0.14	0.463	0.30	0.106	0.443
		3	0.25	0.193	0.48	0.007*	0.130
	LH	1	0.06	0.738	0.31	0.093	0.283
		2	0.17	0.387	0.16	0.386	0.931
		3	0.13	0.508	0.14	0.463	0.832
		4	−0.14	0.458	−0.04	0.820	0.878

Validation of clusters: Task, hemisphere and cluster index refers to the clusters identified in the explorative cohort. Correlation between cortical thickness extracted from these clusters and task scores adjusted for sex, age and education reported with Pearson’s correlation coefficient r and corresponding p-values for healthy controls and MS patients from the validation cohort. The interaction analyses between groups and correlation are reported with p-values from ANOVA * = p < 0.05, § = FDR corrected p < 0.05.

Associations from the explorative cohort can be reproduced in the mildly disabled validation cohort

In our independent validation cohort, we found four SDMT clusters from the explorative analyses being significantly associated with task results. Cluster 2 (p = 0.022) of the left hemisphere is located on the lateral fusiform gyrus - a region which seems to play an important role in recognizing face-like features (Meng et al., 2012). In the right hemisphere, we replicated the correlation for intraparietal sulcus (cluster 2, p = 0.026) involved in arithmetic tasks (Dastjerdi et al., 2013), the superior temporal sulcus (cluster 4, p = 0.032) important for speech perception (Poeppel and Hickok, 2007) and cluster 5 (p < 0.001, pFDR < 0.05) in the parieto-occipital sulcus which showed associations with working memory processing of objects (Poeppel and Hickok, 2007). Similar, two clusters of the right hemisphere associated with VLMT verbal learning could be confirmed in the independent cohort of RRMS patients: Cluster 1 (p = 0.009) is located in the lingual gyrus, which has been identified as involved in word recognition (Mechelli et al., 2002), while the location of cluster 3 (p = 0.007) in the parieto-occipital sulcus indicates an association with working memory processing (Poeppel and Hickok, 2007). Out of the three clusters associated with RWT_animals in the explorative cohort, we found a correlation for cluster 1 (p = 0.030) in the posterior part of the left superior frontal gyrus where highest levels of executive processing lead to activation during working memory tasks (Boisgueheneuc et al., 2006). However, within this small sample we were not able to proof the specificity for MS by ANOVA (Table 7).

## 4 Discussion

Cognitive impairment is a common complaint in MS and has been found to correlate with cortical atrophy. Here, we analysed the association between cortical thickness and performance for an extensive battery of cognitive tests in a representative cohort of MS patients. We obtained several cortical clusters in which cortical thickness correlated significantly with cognitive performance. These clusters were scattered across the cortex; however, the majority of clusters were located in lateraloccipital, parietal and middletemporal areas. In contrast to several task specific associations, i.e. one task, one region, a couple of regions were associated with up to five tasks. They seem to represent regions with a fundamental function needed for several tests such as language processing in the superior temporal sulcus (Poeppel and Hickok, 2007). Importantly, we were able to proof the feasibility of our approach in an independent sample of MS patients with less cognitive impairment. We could further show that the observed association were rather a pathophysiological dependency than a physiological feature, as we did not find the same associations in a group of healthy controls. Our results refine, validate and extend the heterogenous knowledge from previous studies on single tasks or composite cognition endpoints such as we provide direct comparison of task locations in a single, relatively large and representative cohort.

### 4.1 Spatial distribution of associations and functional relevance

We found cognitive performance to be associated with cortical thickness in three main areas in our comparably large sample of MS patients when lesion volume was included in our analyses: the middle temporal regions, parietal regions and the lateral occipital complex.

**Temporal areas.** The observed relationship between cognitive impairment and cortical thickness in temporal areas (superior, middle- and transversetemporal locations) is in line with findings reported by Tillema et al. (Tillema et al., 2016) who identified significantly more temporal thinning in cognitively impaired as compared to cognitively preserved MS patients. There is evidence that the superior temporal gyrus integrates information into a decision making strategy (Paulus et al., 2005), which could explain why cortical thickness in this region was associated with different cognitive subdomains. The temporal pole has been suggested to be a multimodal area associated with semantic processing (Snowden et al., 2004), whereas the fusiform region has been related to high-level visual perception, such as face, object and word recognition, and reduced grey matter density of the area has been linked to dyslexia (Kronbichler et al., 2008). The multimodal functional relationship of temporal regions is mirrored by our results and support the reliability of our approach. We observed associations with mainly language related tests from the domains of attention (e.g. SDMT and TMT-A), memory (digit backwards and VLMT) and executive functioning (both RWT subtests) while there was an only association with spatial processing. Moreover, compared to healthy controls we identified an overlap with atrophic temporal regions in our MS cohort supporting the interpretation of a pathophysiological driven dependency.

**Parietal Areas** (supramarginal gyrus, inferior and superior parietal lobule). The superior parietal lobe was associated with different tasks from the domains of attention/information processing, memory and spatial processing in our cohort. Such a wide spread association indicates an integrative or managing function of the identified regions. This is supported by previous research showing that superior parietal regions are involved in spatial orientation (Corbetta and Shulman, 2002), receive visual and sensual input, and integrates sensory information among different modalities. Thinning of the parietal lobe has been found to occur later in disease progression and to be more prominent in patients who had been diagnosed with both relapse-remitting MS (RRMS) and cognitive impairment (Calabrese et al., 2010). Here, we could not detect significant atrophy in comparison to healthy individuals. Thus, the associations with cognitive performance might indicate an already existing and functionally relevant mild neurodegneration of these areas. However, this interpretation must be confirmed in longitudinal studies and in cohorts with longer disease duration than our sample.

**Lateral occipital Areas.** The lateral occipital lobe is a part of the brain involved in visual object processing (Grill-Spector and Malach, 2004). Our neuropsychological assessment contained mainly tasks involving a visual component. Hence, it appears likely that impaired visual processing due to occipital/temporal cortex pathology would result in poorer performance on cognitive tasks involving a visual component. Consequently, the observed correlations between lateral occipital clusters and cognitive performance appear to be most likely driven by visual processing skills. In addition to these three main areas, clusters were also seen in the isthmus cingulate and posterior cingulate. Similar to parietal areas, the associations were not associated with significant thickness loss if compared with healthy individuals. However, due to the fundamental functional relevance of visual processing, even mild cortical thickness loss might have a clinically relevant impact on cognitive performance.

### 4.2 The pattern of associations overlaps with rich club regions

Interestingly, the regions we observed relate to rich club regions identified by van den Heuvel and Sporns (van den Heuvel and Sporns, 2011). They defined rich club regions as brain regions, which are highly connected and highly central and have an important role in information processing. All of the rich club regions reported by van den Heuvel and Sporns showed correlations with cognitive performance in the present study, namely the precuneus, superior parietal and insular cortex. Steenwijk et al. (2016) reported similar regions in which cortical atrophy was related to cognition in general, major differences being that they did not observe an association for parietal regions.

### 4.3 Rather short disease duration and relapsing disease course might explain the dominance of temporal regions

The fact that we found a rather weak correlation between cortical thickness in the superior frontal gyrus and performance on several neuropsychological tests may seem surprising, given that numerous previous studies have provided evidence for an association between cortical atrophy in the superior frontal gyrus and cognitive deficits in MS patients (e.g. Steenwijk et al., 2016). The relationship between cortical thickness in the superior frontal gyrus and cognition in MS might be mediated by MS type. A study by Riccitelli et al. (Riccitelli et al., 2011) compared atrophy patterns of cognitively impaired and cognitively preserved patients of each MS type and identified atrophy in temporal and occipital regions to correlate with cognitive decrements in RRMS, while the differences between cognitively impaired and cognitively preserved SPMS patients additionally involved atrophy in the frontal lobe, e.g. superior frontal gyrus and middle frontal gyrus. Thus, an association between cortical atrophy in the superior frontal gyrus and cognition may be more likely to be detected in SPMS. Further differences between the studies by Riccitelli et al. (Riccitelli et al., 2011) and Steenwijk et al. (Steenwijk et al., 2016) and the present study were a longer disease duration of patients in these studies, a different methodological approach addressing patterns of atrophy instead of raw cortical thickness values in our study and that these studies compared overall cognitive performance and that Steenwijk et al. (Steenwijk et al., 2016) had a large sample size and compared their patients against a healthy control group. Taken together, the observed regional associations show an overall functionally meaningful spatial distribution that confirms and extends previous research to a broader spectrum of specific cognitive tasks.

### 4.4 Reliability and specificity of regional associations

We could further support reliability and dependency with MS for some of our results by applying our findings from the explorative cohort to a second cohort which included a subgroup of mildly impaired RRMS patients and a subgroup of healthy controls. However, the sample size was too small to properly validate the explorative results. With exception of one cluster that was correlated with SDMT performance in the explorative and healthy control cohort, we observed for each cognitive domain at least one clusters in the RRMS group only. Thus, cortical thickness of those clusters might be a putative disease-driven correlate of task-specific cognitive performance in our study populations. However, based on the cross-sectional data set it cannot be answered how and when MS-specific atrophy development contributes to the observed associations. Recent research indicates that atrophy in MS follows distinctive spatio-temporal patterns with a pronounced influence of cortical damage on cognitive dysfunction in later disease stages (Eijlers et al., 2018). As our cohort has rather short disease duration and represents mainly RRMS patients, our findings must be regarded as snapshot of the early disease and the findings cannot easily be transferred to cohorts with longstanding MS. Our results may be particularly useful for future MRI studies, as our task- or domain-specific clusters maybe applied as masks functional or structural MRI outcomes in interventional studies addressing specific cognitive domains in comparable cohorts. The lack of associations in healthy controls supports a disease specificity of our results but the sample size is too small to exclude a weaker association. Moreover, cortical thickness correlations might not be the most suitable method for localisation of such functions in healthy individuals.

### 4.5 The association between cortical thinning and cognitive performance cannot be explained by lesions alone

Lesion volumes were found to be related to cognitive impairment in MS patients (e.g.(Patti et al., 2009)), but there is also evidence for cortical atrophy and lesions to contribute independently to the presence of cognitive deficits(Amato et al., 2007). More recently, the relevance of white matter integrity for cognitive decline has been demonstrated for early RRMS patients (Eijlers et al., 2018). The present study identified a range of brain regions in which cortical atrophy was significantly correlated with decreased cognition and the number of brain regions was substantially lower when lesion volumes were added as an additional covariate. The finding that some associations were seen after effects of lesion volume were discarded from the analyses is meaningful in the sense that cortical atrophy appears to hamper cognition independently of potential lesions in these areas. However, the causal relation between white matter lesions, regional atrophy and cognition should be addressed in future research. For example, DTI data that are not available in our dataset would allow defining local dependencies based on tractography. Unfortunately, we were not able to analyse the impact of cortical lesions in our study. They have been identified as highly important for disability and cognition in MS (Magliozzi et al., 2018). However, their detection in a clinal setting is not yet easy to accomplish and might rather be a domain of ultra-high field imaging (Faizyet al., 2017, Harrisonet al., 2015).

## 5 Limitations

A downside of the present study is that, due to the retrospective nature, complete records of previous medical treatments were not available for all patients included in the study. However, considering the sample size of the study, there is no good reason to assume systematic effects of medical treatment that may have mediated our results. Future studies should nevertheless explore whether medical treatment unrelated to MS may affect cognitive performance and could be a potential confound when assessing relationships between cognitive performance and cortical atrophy. In addition, our findings are limited by putative training effects that might explain for example the lack of finding for the PASAT. Moreover, nearly a fourth of our cohort declined performing the PASAT indicating a probable selection bias towards cognitively preserved patients due to the high stress level of the task. It is also important to note that MRI scans and neuropsychological assessments were up to several months apart. Although it does not seem very likely, neuropsychological profiles and/or cortical thickness measures may have changed substantially within one year. Moreover, we cannot exclude for sure that a relapse occurred between the neuropsychological test and the MRI which might have reduced the sensitivity of findings. Ideally, future trials should conduct MRI examinations and neuropsychological assessments within a short time window. Moreover, there are some technical aspects that might have influenced our results: Scan-rescan reproducibility of segmentations was not investigated and variability due to segmentation cannot be estimated. However, we performed manual corrections for all segmentations which excludes major segmentation errors and increases the specificity of cortical segmentation (Popescu et al., 2016). While there are several pipelines for cortical or grey matter analysis, we used here the FreeSurfer pipeline for the following reasons: It’s widespread use allows an easier replication and use of our freely distributed association maps. Moreover, its cortical thickness estimates are more reliable than other pipelines (Popescu et al., 2016) and surface based analyses seem to have some advantages compared to voxelwise methods (Clarkson et al., 2011). As a consequence, we applied only surface based correlations between cortical thickness and cognitive performance that did not address the relevance of other structures, namely deep grey matter and white matter tracts (Eijlers et al., 2018). In addition, we did not correct for global atrophy and could thus not proof a distinctive pattern of associations which restricts the comparability with previous research (Steenwijk et al., 2016). Resting state functional connectivity might be a promising extension for future studies, as it has been observed that changes in grey matter volume are often accompanied by altered resting state functional connectivity between these areas and other brain regions (Giliet al., 2011, Liaoet al., 2011, Luiet al., 2009). Future studies could improve closeness to the underlying pathophysiology by new approaches such as sodium MRI, which has recently been identified as a promising measure to detect associations between tissue damage and cognition in MS (Maarouf et al., 2017). Furthermore, the cross-sectional design of the present study does not allow a causal interpretation of the results and we cannot determine if the regions found really atrophied or not. Longitudinal studies will be required to confirm a causal link between cortical atrophy and cognitive impairments (see (Eshaghi et al., 2018)). Moreover, the small and selective validation cohort is not sufficient to confirm our explorative findings and the lack of association in healthy controls might as well be a sample size effect. However, most association studies do not provide any validation strategies and even if our approach is not confirmatory we believe that the findings strengthen a pure explorative approach. Finally, providing our results as freely available atlas will hopefully lead to validation by independent groups. As the present sample consisted primarily of patients with RRMS and only a small subset of patients with a progressive MS type, future research should aim at identifying potential differences in relapsing-remitting and progressive MS types.

## 6 Conclusion

Summarized, the well-known heterogeneity in MS symptoms and disability accumulation is also represented by locally heterogeneous but task specific distribution of cortical regions associated with several cognitive functions in our cohort. Our findings support the concept of spatially segregated neurodegenerative processes in MS and provides an informative spatial pattern for a broad range of cognitive tasks in a representative cohort of early MS. We believe that our results may prove useful in diagnosis and rehabilitation of cognitive impairments and may serve as guidance in future magnetic resonance imaging (MRI) studies.

## CRediT authorship contribution statement

**Jan-Patrick Stellmann:** Conceptualization, Methodology, Investigation, Formal analysis, Writing - review & editing. **Nadine Wanke:** Conceptualization, Methodology, Investigation, Writing - original draft, Formal analysis, Writing - review & editing. **Adil Maarouf:** Methodology, Writing - review & editing. **Susanne Gellißen:** Methodology, Investigation, Writing - review & editing. **Christoph Heesen:** Conceptualization, Methodology, Investigation, Writing - review & editing. **Bertrand Audoin:** Conceptualization, Methodology, Writing - review & editing. **Stefan M. Gold:** Conceptualization, Methodology, Writing - review & editing. **Wafaa Zaaraoui:** Conceptualization, Methodology, Writing - review & editing. **Jana Poettgen:** Conceptualization, Methodology, Investigation, Writing - review & editing.

## Declaration of Competing Interest

The authors declare that they have no known competing financial interests or personal relationships that could have appeared to influence the work reported in this paper.
