# Supplementary material for: Cognitive performance shows domain specific associations with regional cortical thickness in multiple sclerosis
Source: Neuroimage Clin. 2021 Feb 24;30:102606. doi: 10.1016/j.nicl.2021.102606 (PMC7985400; doi:10.1016/j.nicl.2021.102606)
Supplement: Supplementary Data 1 [file mmc1.pdf]

Cognitive performance shows domain specific associations with regional cortical thickness in multiple sclerosis

Jan-Patrick Stellmann, Nadine Wanke, Adil Maarouf, Susanne Gellißen, Christoph Heesen, Bertrand Audoin, Stephan M. Gold, Wafaa Zaaraoui, Jana Poettgen

Appendix

Detailed description of each task used in the neuropsychological assessment, their individual association maps and a brief review of the literature concerning previous finding is provided below.

ATTENTION

Test battery of Attentional Performance

Test battery of Attentional Performance (TAP). We employed three subtests of the battery to evaluate attentional capacities. The alertness task requires speeded key-press responses when a cross is presented in the middle of the screen. The task comprises two conditions, one without warning tone to assess tonic alertness, and another one in which a warning tone is presented before the cross appears on the screen to assess the phasic component of alertness. The selective attention task (Go/NoGo) which asks for speeded key-presses to only one out of two visually distinctive types of crosses successively presented on the screen. The divided attention task (dual task) which requires parallel processing of visually and auditory presented targets. Two tones, high and low in pitch, are alternating; one task is to press a key as fast as possible if the same tone is presented two times in succession. The second task is to monitor moving dots and crosses in fixed places on the screen and to press a key each time four crosses form a small square.

Our findings (figure A1) are in line with the well-established dominance of the right human brain hemisphere in attention allocation (Cai et al., 2013). Divided attention can target the same modality (within-modal tasks) or different modalities (cross-modal tasks). To identify brain areas involved in distributing and dividing attention between tasks independently of the respective modality involved, Vohn et al. (2013) conducted a conjunction analysis of functional MRI (fMRI) data of within- and cross-modal paradigms. The authors reported a right-hemisphere activation network including the precentral gyrus, the superior frontal gyrus, the claustrum and the inferior and superior parietal lobule. This fits well with the observed cluster in the superior parietal cortex, which suggests that the superior parietal cortex

is critically involved in divided attention in MS patients. Analysis II revealed a cluster in lateral occipital cortex. This can be explained by the fact that one of our tasks involved the visual modality, as the lateral occipital cortex is a brain structure known to be engaged in visual object processing (Grill-Spector & Malach, 2004).

Figure A1 – Cortical regions associated with TAP divided attention

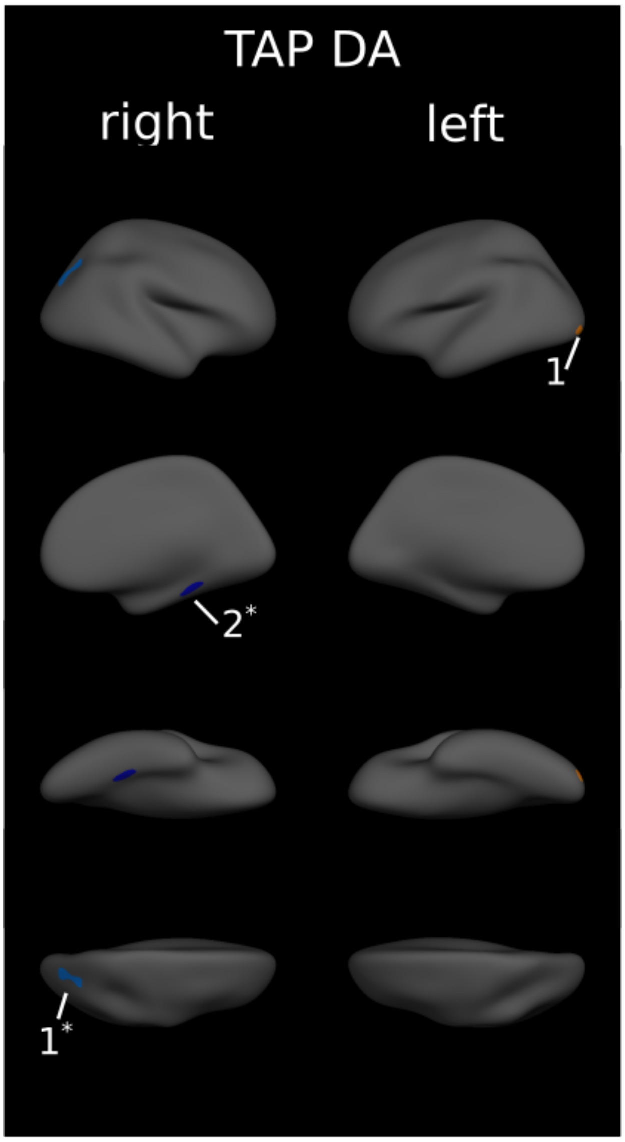

Clusters of association between cortical thickness and TAP divided attention displayed on the FreeSurfer fsaverage surface. Numbers correspond to cluster index in table 4.

SDMT

The Symbol Digit Modalities Test (SDMT) - oral version - was used to assess information processing. The substitution task involves a reference key, which pairs specific numbers with geometric figures. Examinees are instructed to work through a series of geometric figures by reading out the respective corresponding number, being as fast and accurate as possible.

Our findings (figure A2) are in line with results of earlier studies, which have related SDMT performance to activation patterns and cortical pathology across the brain (Forn et al., 2011; Louapre et al., 2016). This may reflect the fact that the SDMT requires several cognitive capacities, such as divided attention (Ponsford & Kinsella & 1992), complex visual scanning and tracking (Shum, McFarland, & Bain, 1990), and perceptual speed and memory (Laux & Lane, 1985; Lezak, 1995). In a longitudinal study, a decline in SDMT performance correlated with decreased volume in the caudate nucleus in MS patients (Loitfelder et al., 2014), whereas cross-sectional studies found SDMT scores in MS to be associated with GM volume in frontal and temporal clusters (Nocentini et al. 2014) and cortical thickness in bilateral frontal lobe (Calabrese et al., 2010). A study by Wen et al. (2015) failed to observe a correlation between SDMT performance and regional age-adjusted grey matter volume; however, when they correlated SDMT scores with age-adjusted transverse relaxation rates, another MRI measure that can be employed to quantify the decay of cerebral tissue, they detected several correlations between SDMT scores and brain structures in different areas, among them mainly frontal and temporal brain regions such as the superior temporal gyrus, partially overlapping with our results.

Figure A2 – Cortical regions associated with SDMT

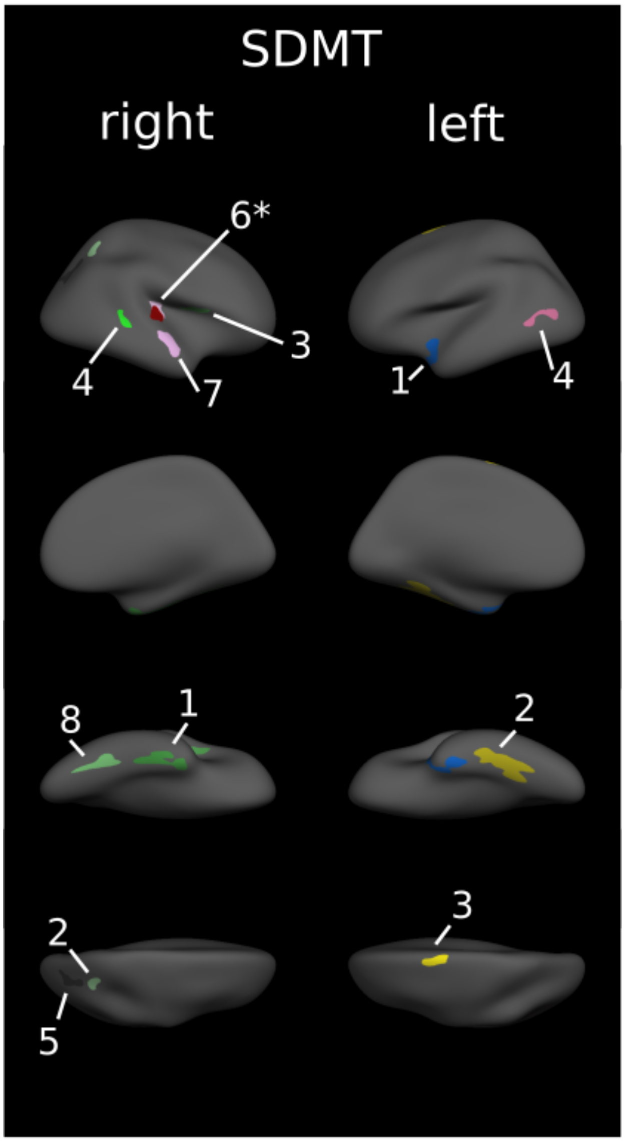

Clusters of association between cortical thickness and SDMT displayed on the FreeSurfer fsaverage surface. Numbers correspond to cluster index in table 4.

TMT-A

Trial Making Test A (TMT-A) was employed to evaluate information processing speed. The task involves linking numbers from 1 to 23 randomly scattered on a sheet of paper with a pencil in an ascending order as fast as possible.

Similarly to our results (figure A3), Achiron et al. (2012) reported cortical thickness of the left superior temporal sulcus to be associated with information processing speed in MS patients. Using fMRI, Smith et al. (2012) observed an increase of neuronal activation in the right prefrontal cortex and superior

temporal gyrus and a decrease in the left middle temporal and superior temporal gyri in MS patients as compared to the healthy control group while they performed an information processing speed task.

Figure A3 – Cortical regions associated with TMT-A

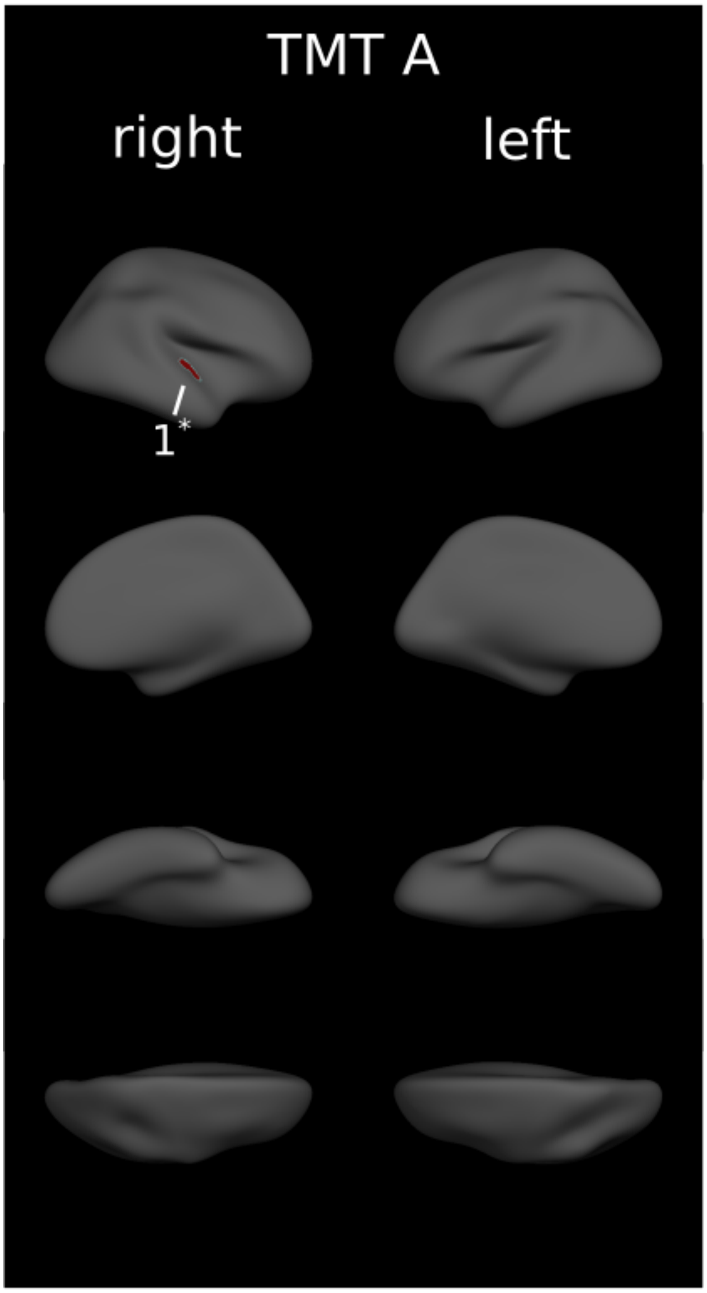

Clusters of association between cortical thickness and TMT-A displayed on the FreeSurfer fsaverage surface. Numbers correspond to cluster index in table 4.

PASAT

The assessment further included the Paced Auditory Serial Addition Test (PASAT) tapping into divided attention, working memory and numeracy skills. The task comprises a stream of auditory presented numbers and the examinee is asked to always add the two last numbers of the stream, say the sum out loud and to continue with the addition of the next auditory number and the one that was presented before. The lack of associations in our cohort is in line with a study by Nocentini et al. (2014) who also failed to observe a correlation between regional cortical thickness and PASAT performance. In contrast to this, an earlier study reported a relationship between PASAT scores and GM volume in the prefrontal cortex, precentral gyrus and superior parietal lobe bilaterally as well as in the left precuneus and right cerebellum (Morgan, 2006). While the study by Nocentini et al. (2014) and the present study used a PASAT version with interstimulus intervals of 3 seconds, Morgen et al. (2006) used considerably shorter intervals between stimuli (1.8 seconds on average). Moreover, the patients investigated by Morgen et al. (2006) had shorter disease durations than those of the two other studies. Thus, subtle impairments in task performance might relate to well localized areas, whereas more severe impairments might be associated with a more widespread pattern of tissue damage. Performance on the PASAT relies on a variety of cognitive subdomains (divided attention, working memory and calculating capacity) and deficits in task performance may be reflected in damage of many different neuroanatomical structures and/or network properties of the brain. Studies using diffusion tensor imaging (DTI) suggest that PASAT performance is indeed related to specific brain network measures. For instance, Yu et al. (2012) reported that a summary measure of microstructural integrity (FA value) in different brain regions within the striatum, thalamus, corpus callosum and external capsule was significantly associated with PASAT performance. Similar findings have been described by Dineen et al. (2009) and Roca et al. (2008). The PASAT is a rather difficult task, which is stressful to perform for many participants. For that reason, data is not always available for the task, which left us with a substantially smaller subsample (N=75). Hence, a potential lack of power and stress-related performance decrements could be considered as alternative explanations for the lack of significant associations between performance and cortical thickness.

MEMORY

Digit span

The Wechsler number digits forward /backward task. A stream of numbers varying in length is read out loud to the examinee who is supposed to repeat the numbers in the same order (forward; short term memory) or in the opposite order (backward; working memory).

Richardson and co-workers (2011) investigated grey matter density in normal and dyslexic patients and found the left posterior superior temporal sulcus to correlate with both performance in the digit forward and digit backward task. The superior temporal gyrus is part of the primary auditory cortex. Given that the perception of auditory information is essential for subsequent processing and consolidation processes when performing the task, our findings (figure A4) provide evidence for an association between cortical thickness of the superior temporal gyrus and digit backwards performance in MS patients. Cortical thickness in bilateral lateral occipital clusters was also correlated with performance on the working memory task. The lateral occipital cortex is known to be involved in visual object processing (e.g. Grill-Spector & Malach, 2004). Thus, it may seem surprising to detect a correlation between cortical thickness and performance on a task that does not require visual processing. A potential explanation for this finding could be that visual mental imagery of the auditorily presented numbers may be a helpful strategy to improve performance on the task, given that previous research observed activation in early visual cortex during visual mental imagery (Kosslyn et al., 1993; Kosslyn & Thompson, 2003). This idea is supported by findings from Gerton et al. (2004) who reported fMRI activation in visual processing areas while participants performed the digit span backward task when no visual stimuli were present.

Figure A4 – Cortical regions associated with digit span backwards

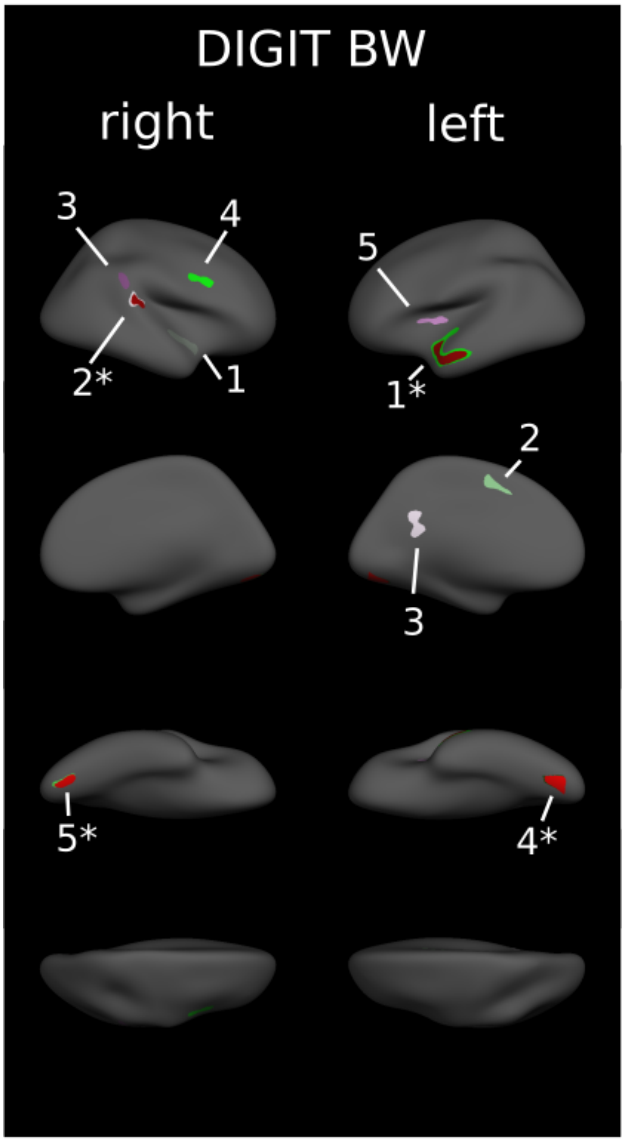

Clusters of association between cortical thickness and digit span backwards displayed on the FreeSurfer fsaverage surface. Numbers correspond to cluster index in table 5.

Wechsler VLM

The Wechsler verbal logical memory task in which one is to repeat as many details as possible of a short story directly after hearing the story and 30 minutes later (delayed recall).

We observed a correlation of verbal memory and cortical thickness in the right hemisphere (figure A5), which stands in contrast to the well-known left hemispheric lateralization for language processing. The anatomic location of the fusiform gyrus is close to the lingual gyrus, and apart from the well established role in face processing and visual recognition (Grill-Spector & Malach, 2004), the structure has been linked to dyslexia (Koyoma et al., 2013). Thus, general literacy may contribute to

performance on the delayed version of the verbal-logical memory task by facilitating encoding and retrieval of verbal information

Figure A5 – Cortical regions associated with VLM delay

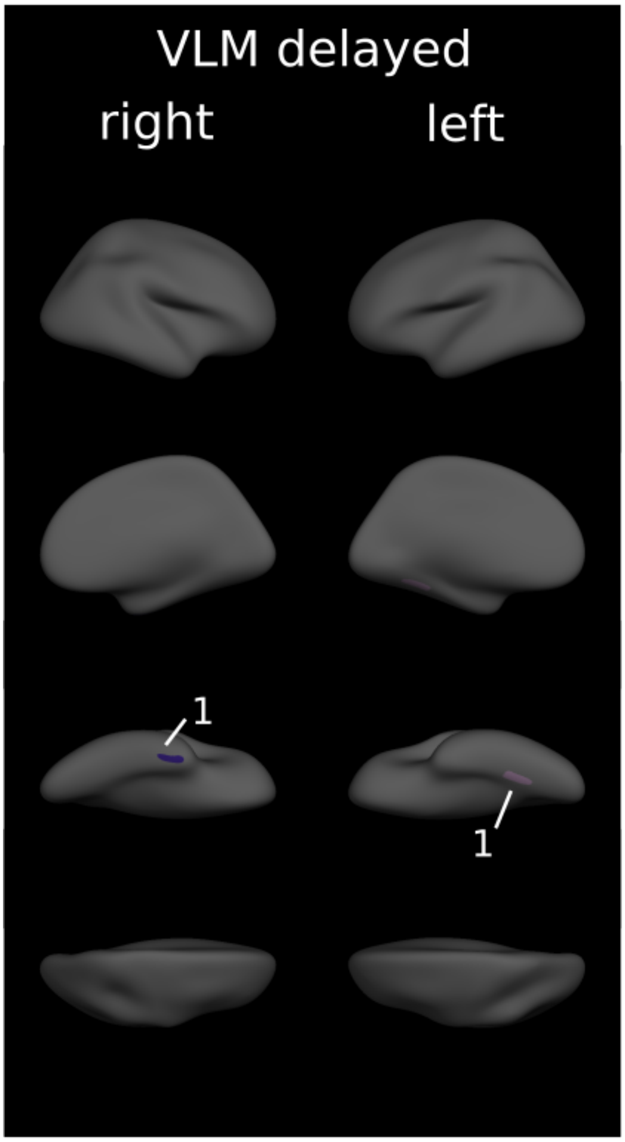

Clusters of association between cortical thickness and VLM delay displayed on the FreeSurfer fsaverage surface. Numbers correspond to cluster index in table 5.

REY recall

The Rey-Osterrieth complex figure test evaluates spatial constructive abilities when examinees are asked to copy a complex geometrical figure and assesses figural memory when the figure is to be drawn from memory 30 minutes later.

Rey-Osterrieth figure – delayed recall: Performance on this figural memory task correlated with clusters located across both hemispheres of the brain (figure A6). None of the clusters remained with lesion volume as an additional covariate. The delayed recall of the Rey-Osterrieth figure relies mainly on visuo-spatial memory within declarative memory, which has been associated with the hippocampus and other anatomically connected regions in the right temporal lobe (Shin et al., 2006). Tillema at al. (2016) associated visual memory with parietal thinning. Although the different conditions of the Rey-Osterrieth figure (copy, recall) theoretically enable separate assessments of encoding, storage and retrieval processes (Shin et al., 2006), it should be noted that performance in the recall condition relies substantially on previous completion of the copy condition, which involves attention and concentration, visuo-spatial perception, visual- motor coordination, and executive skills, such as planning and organizational skills. Whereas visuo-spatial perception involves the occipital-parietal (dorsal) pathway and object analysis requires the occipital-temporal (ventral) stream (Mishkin et al., 1982), executive functioning, for instance, is related to occipital-parietal and prefrontal regions. Thus, it may not come as a surprise that we observed clusters scattered across the brain that appeared related to figural memory performance (Lezak, 1995; Ogden-Epker & Cullum, 2001).

Figure A6 – Cortical regions associated with Rey figure recall

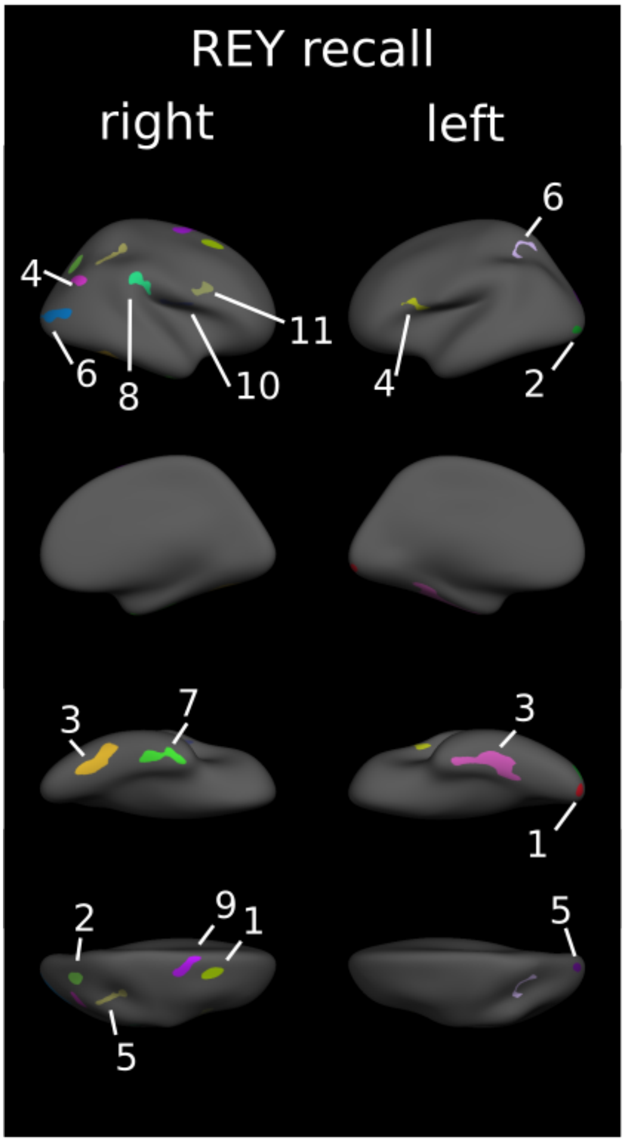

Clusters of association between cortical thickness and Rey figure recall displayed on the FreeSurfer fsaverage surface. Numbers correspond to cluster index in table 5.

VLMT

The verbal learning and memory task (VLMT) involves a word list of 15 words being read out to the examinee several times who is supposed to repeat all the words to evaluate supraspan, verbal learning, verbal memory, and recognition.

Verbal learning and memory task. There were no clusters associated with the number of words that were remembered after the word list was read out for the first time (supraspan). Verbal learning ability was correlated with a cluster in the left hemisphere (figure A7), extending over the dorsal part of the cingulum, subparietal sulcus and precuneus. Verbal memory scores were not associated with any clusters in the brain. Using the English version of the task used in the present study, Walhovd et al.

(2006) failed to identify meaningful clusters between cortical thickness and verbal memory after 30 minutes, but did observe effects of cortical thickness when the recall was delayed by weeks or months. Thus, it seems possible that verbal memory performance after 30 minutes is too short to observe a correlation with cortical thickness.

Recognition performance for learned verbal material was not correlated with any brain regions, however, after controlling for lesion volume, an association with a cluster in the superior parietal cortex of the right hemisphere was detected (figure A8). A study by Nocentini et al. (2014) found associations between verbal memory and GM volume clusters in frontal and temporal areas (left frontal pole; right precentral, postcentral and supramarginal gyrus; right central opercular cortex, planum polare and insular cortex). Schlund and Cataldo (2007) reported main effects of recognition in various clusters using an fMRI task, among them the right cuneus and right superior parietal lobule, which is in line with our results. In summary, this indicates cortical thickness of the superior parietal lobule to be associated with verbal recognition performance; it should, however, be noted that further brain regions might be related to verbal recognition in MS, which the present study was unable to detect.

Figure A7 – Cortical regions associated with VLMT verbal learning

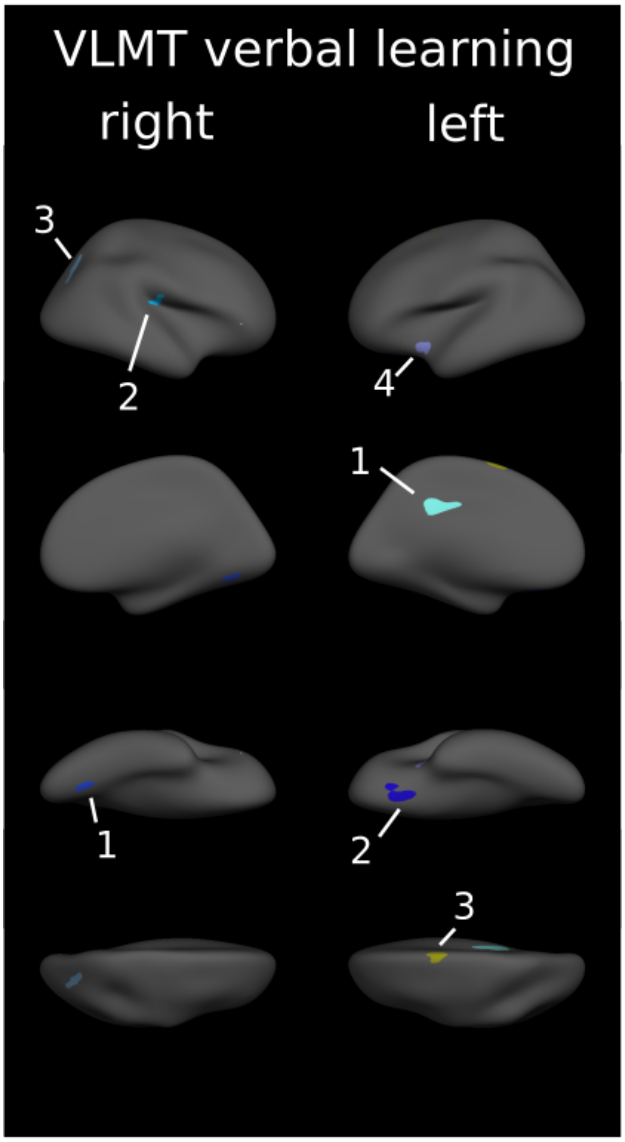

Clusters of association between cortical thickness and VLMT verbal learning displayed on the FreeSurfer fsaverage surface. Numbers correspond to cluster index in table 5.

Figure A8 – Cortical regions associated with VLMT recognition

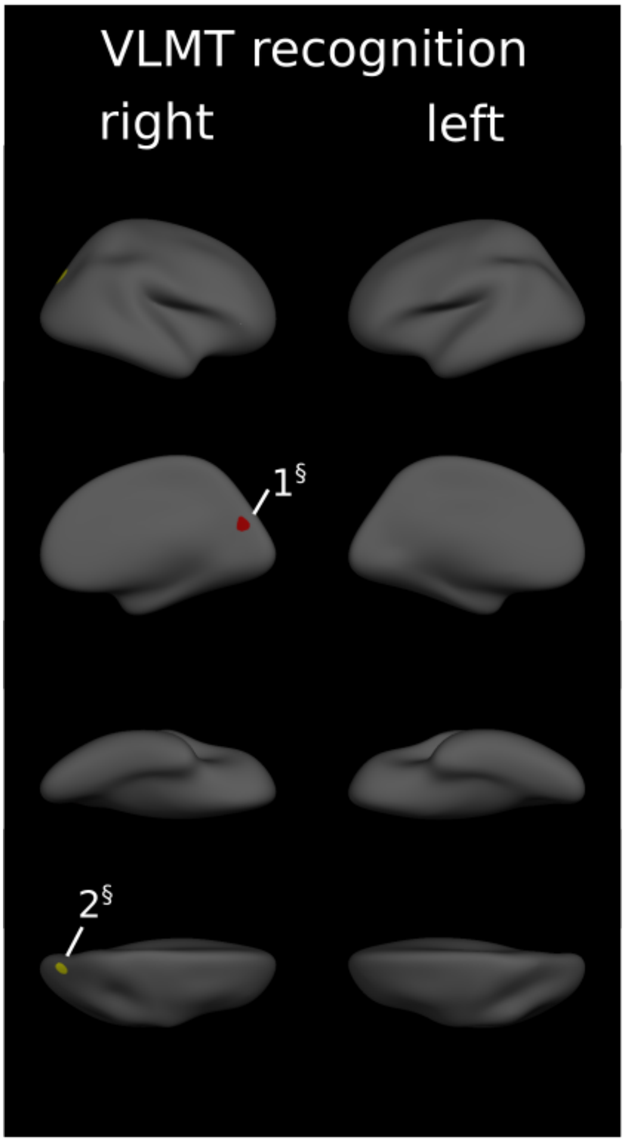

Clusters of association between cortical thickness and VLMT recognition displayed on the FreeSurfer fsaverage surface. Numbers correspond to cluster index in table 5.

SPATIAL PERCEPTION

LPS7

The Leistungsprüfungssystem (LPS) 7 was used to evaluate spatial cognitive abilities. The task requires fast detection of mirror-inverted numbers or letters presented among lines of the same numbers/letters, which are not mirror-inverted but may be slightly rotated.

The observed cluster (figure A9) makes sense, as the fusiform region is known to be involved in visual perception (Grill-Spector & Malach, 2004).

Figure A9 – Cortical regions associated with LPS-7

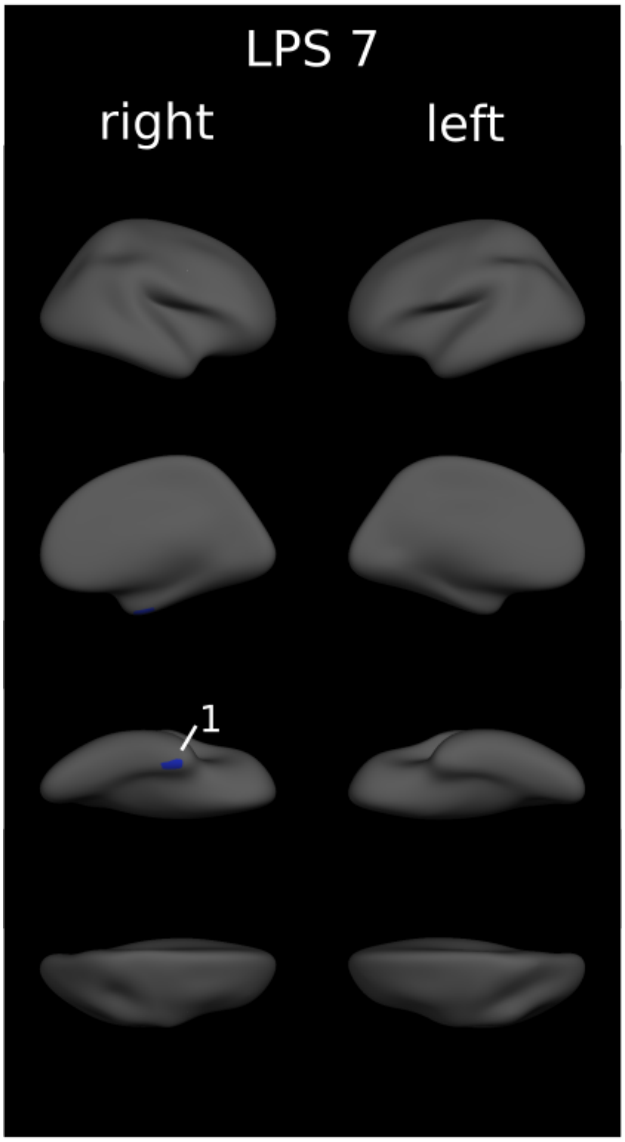

Clusters of association between cortical thickness and LPS-7 displayed on the FreeSurfer fsaverage surface. Numbers correspond to cluster index in table 6.

REY copy

Rey-Osterrieth Figure – copy. The Rey-Osterrieth Complex Figure is a measure of visuoconstruction, suggesting the task may largely involve the right hemisphere. As previously mentioned, the copy condition of the Rey-Osterrieth figure involves several cognitive functions, including attention and concentration, visuo-spatial perception, visual-motor coordination, and executive functions (Shin et al., 2006; Meyers & Meyers, 1995; Trojano et al., 2004; Somerville, Tremont, & Stern, 2000; Watanabe et al., 2005).. Scores on a modified design of the task have been found to correlate with working memory performance in various types of dementia (Freeman et al., 2000), giving rise to the assumption that the frontal cortex may be involved in organizational aspects of the copy task (Melrose, Harwood, Khoo, Mandelkern, & Sultzer, 2013). Cerebral metabolism as measured by positron emission tomography (PET) was found to correlate with a range of cortical clusters in patients with probable dementia during the copy task, among them a large cluster in the right posterior cortex including the superior and inferior parietal lobules, lateral temporal cortex and fusiform and occipital cortex; further correlations were seen in the right prefrontal cortex, right frontal eye fields, right precentral gyrus and left posterior parietal and occipital cortex. Copying an image has been suggested to involve different processes: analysis of the target elements and their spatial relations, preparation of a plan, storage of the plan in short term memory and translation into a motor program, the execution of the plan (drawing), and monitoring deviations (Grossi & Trojano, 1999). A range of neuroimaging studies has identified a number of brain areas that were involved in drawing: the dorsal and ventral visual pathways, as well as premotor/motor areas involved in hand movement (Makuuchi, Kaminaga, & Sugishita, 2003) and prefrontal regions (Harrington, Farias, & Davis, 2009; Miall, Gowen, & Tchalenko, 2009). Models assume that visual information about the object to-be-copied passes through the dorsal visual stream in order to be spatially processed and concurrently passes the ventral visual stream where semantic representations about the target objects may be activated (Harrington et al., 2009; Makuuchi et al. 2003). Planning and self-monitoring during the task is assumed to be reflected in prefrontal regions (Miall et al., 2009).

Figure A10 – Cortical regions associated with Rey figure copy

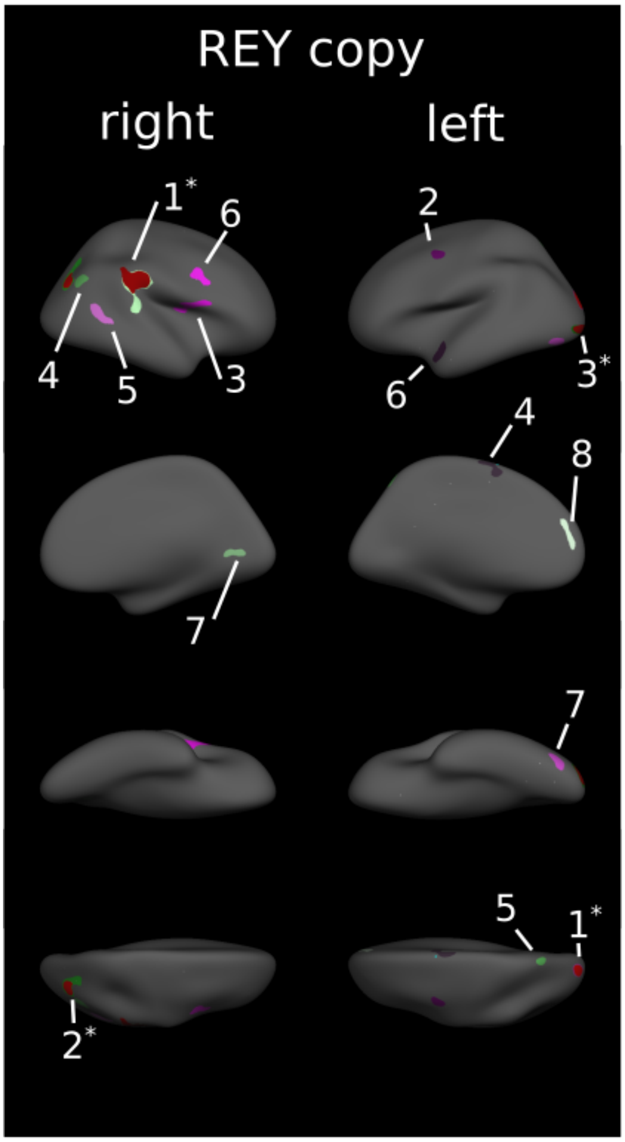

Clusters of association between cortical thickness and Rey figure copy displayed on the FreeSurfer fsaverage surface. Numbers correspond to cluster index in table 6.

EXECUTIVE FUNCTIONING

TMT-B

The Trial Making Test B (TMT-B) was used to evaluate task switching. Numbers and Letters have to be linked in an alternating and ascending order.

The superior temporal gyrus is important for processing auditory information, giving rise to the idea that auditory imagery might help in TMT-B performance. A range of studies have identified the inferior parietal lobule and rostrolateral prefrontal cortex as key areas involved in relational reasoning (Wendelken, Ferrer, Whitaker, & Bunge, 2016; Prado, Chadha, & Booth, 2011; Krawczyk, 2012), one of them being a meta-analysis of more than twenty neuroimaging studies that has established the posterior parietal cortex as particularly important for relational reasoning (Prado et al., 2011). Moreover, a recent study by Schel and Klingberg (2016) has associated the right intraparietal sulcus with mathematical ability. There is good evidence to assume that logical reasoning and mathematical abilities are fundamentally related (Morsanyi & Szücs, 2015), giving reason to suspect that the intraparietal sulci of both hemispheres are involved in logical and mathematical problem solving, with the hemisphere being more involved depending on the exact task properties.

Figure A11 – Cortical regions associated with TMT-B

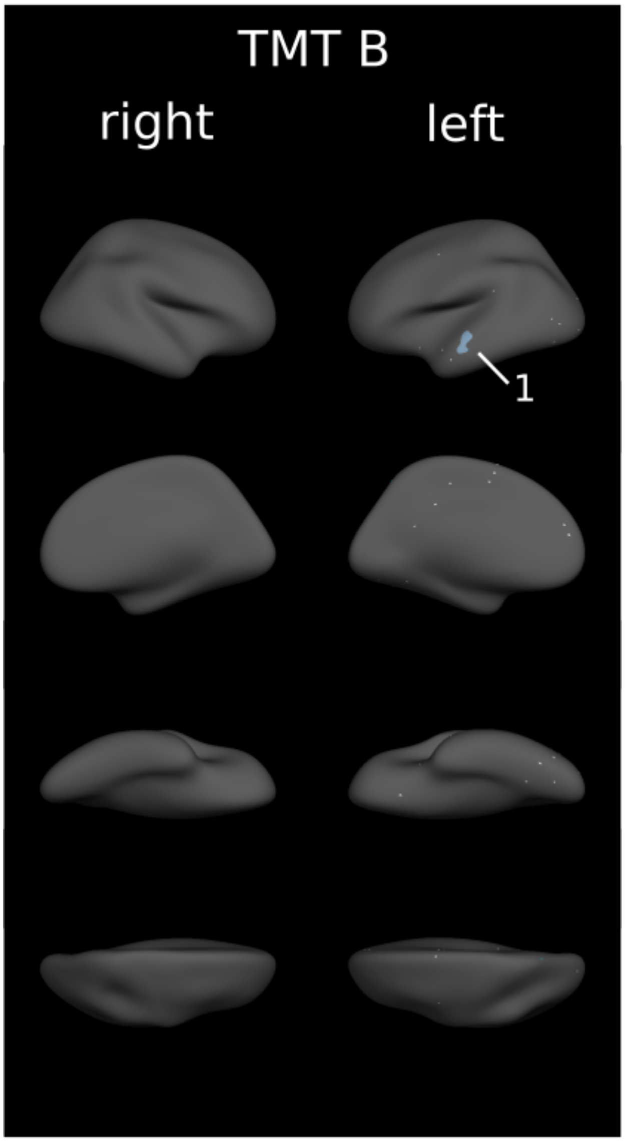

Clusters of association between cortical thickness and TMT-B displayed on the FreeSurfer fsaverage surface. Numbers correspond to cluster index in table 7.

LPS-3

The Leistungsprüfungssystem (LPS) 3 assesses logical reasoning.

Figure A12 – Cortical regions associated with LPS-3

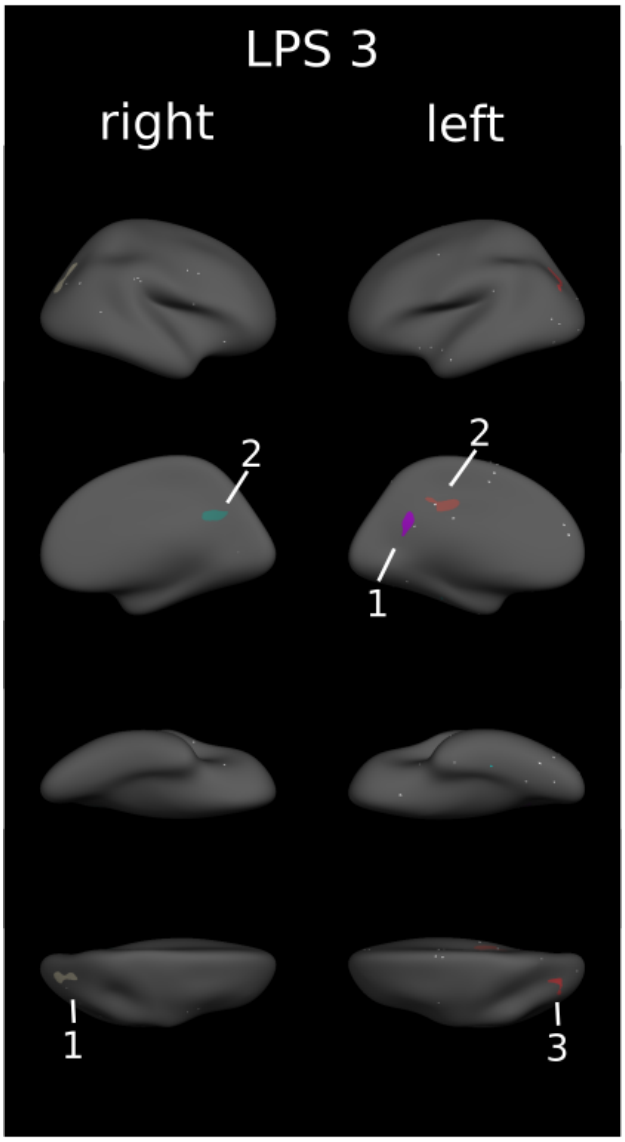

Clusters of association between cortical thickness and LPS-3 displayed on the FreeSurfer fsaverage surface. Numbers correspond to cluster index in table 7.

RWT

The Regensburger Word Fluency Task (RWT). The subtest "animals" (with a test duration of 2 min) was employed to assess word fluency. The task is to enumerate as many words as possible from the category "animals". The subtest G-R aims at evaluating cognitive flexibility; the instruction is to enumerate words beginning with "G" and "R" in an alternating order while repetitions, words beginning with identical word stems and proper nouns are not allowed.

Our results (figures A13 & A14) partially overlap with those observed in earlier studies. A recent study by Geisseler et al. (2016) related verbal fluency as measured by the RWT subtask "S", which requires

fast enumeration of different words beginning with “s”, to left-sided thinning of the anterior cingulate, superior frontal, lateral orbitofrontal, fusiform and superior parietal region.

Figure A13 – Cortical regions associated with RWT animals

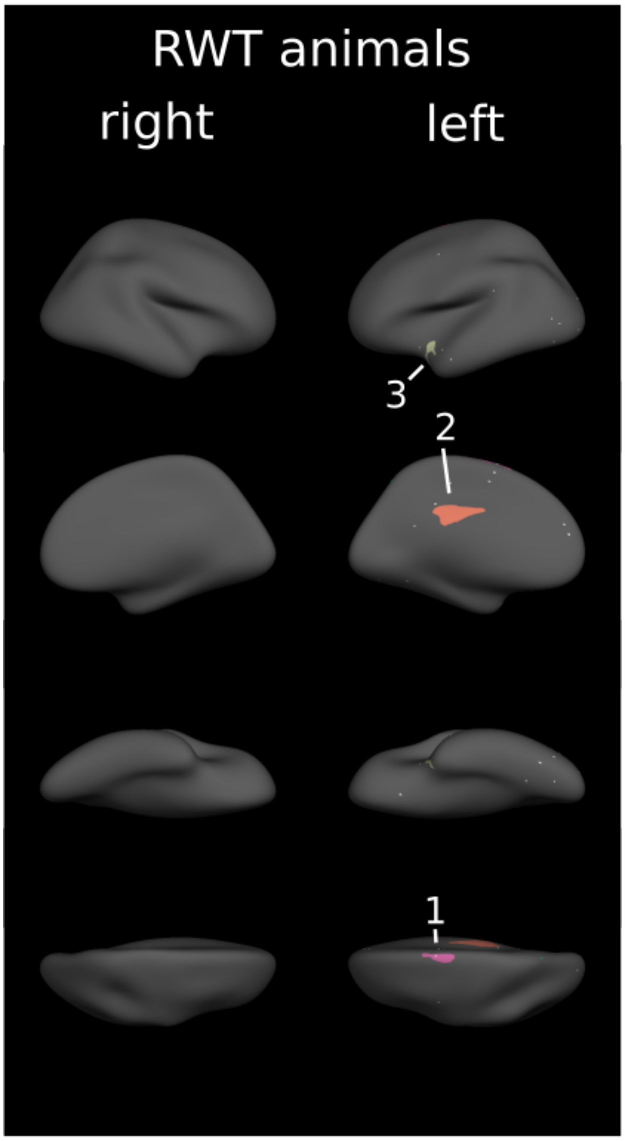

Clusters of association between cortical thickness and RWT animals displayed on the FreeSurfer fsaverage surface. Numbers correspond to cluster index in table 7.

Figure A14 – Cortical regions associated with RWT G/R

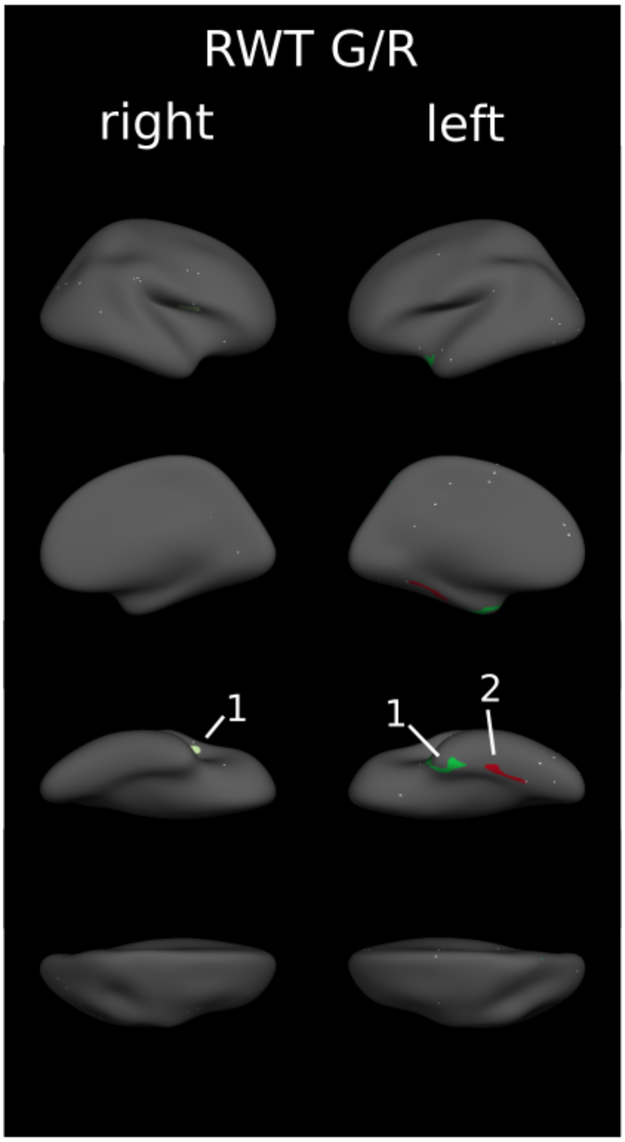

Clusters of association between cortical thickness and RWT G/R displayed on the FreeSurfer fsaverage surface. Numbers correspond to cluster index in table 7.

Zoo Task

The Zoo Map Task aims to evaluate planning skills. The paper-and-pencil task is to plan a route through the zoo while adhering to a list of rules. The task involves to create a plan (high demand condition) and to follow a given plan (low demand condition). Negative penalty scores are added for rule breaks and lack of speed.

A potential reason could be that the total task score comprises subscores of a high demand condition and a low demand condition in equal proportions, thereby possibly skewing the relationship between test scores and level of cognitive impairment.

Figure A15 – Correlation between cortical thickness and SDMT in clusters: controls and RRMS

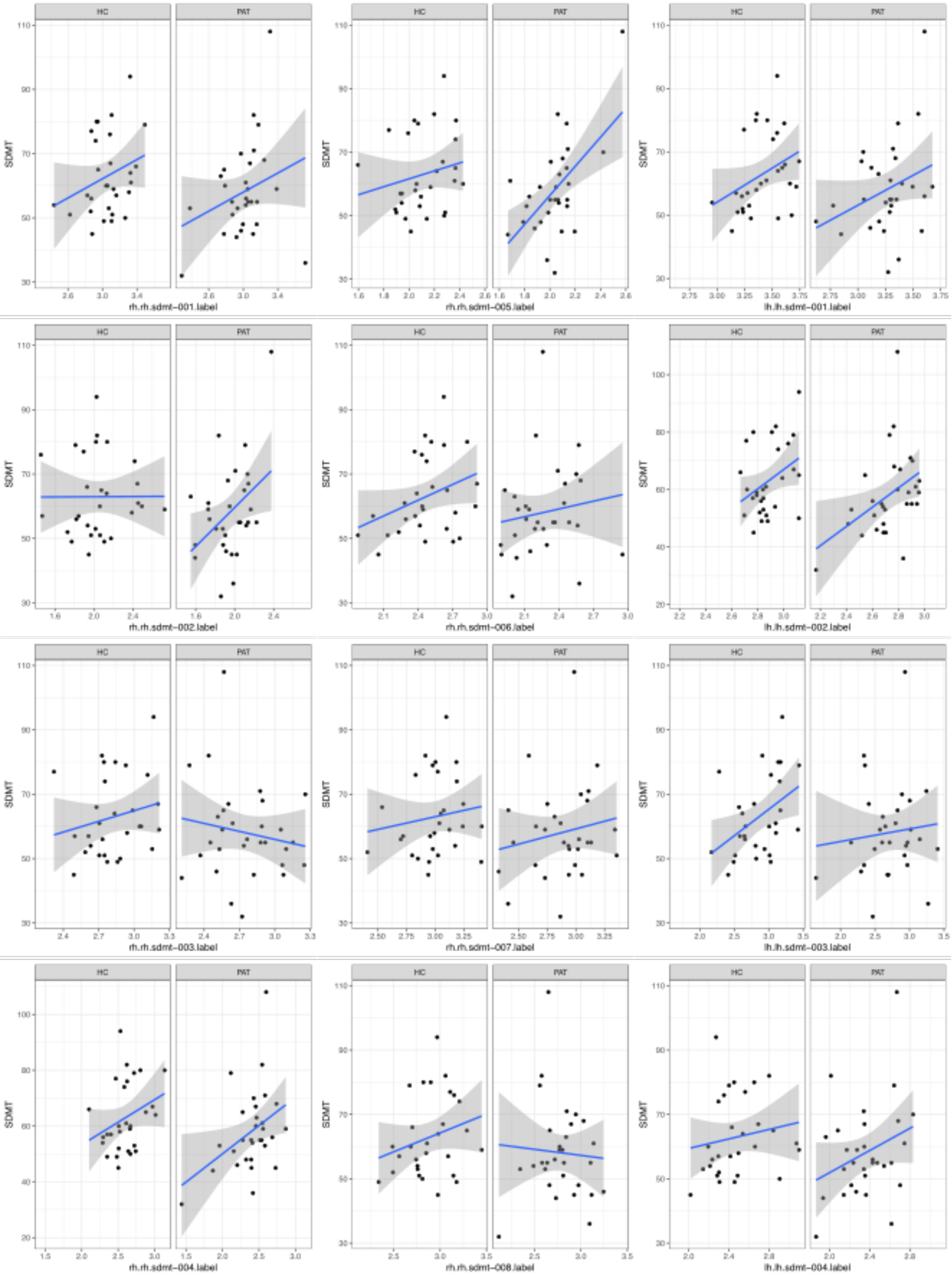

Dotplots with cortical thickness of each cluster and cognitive test performance, regression estimate (blue line) and its 95% confidence interval (grey area). Results for controls on the right, patients on the left

Figure A16 – Correlation between cortical thickness and RWT animals in clusters: controls and RRMS

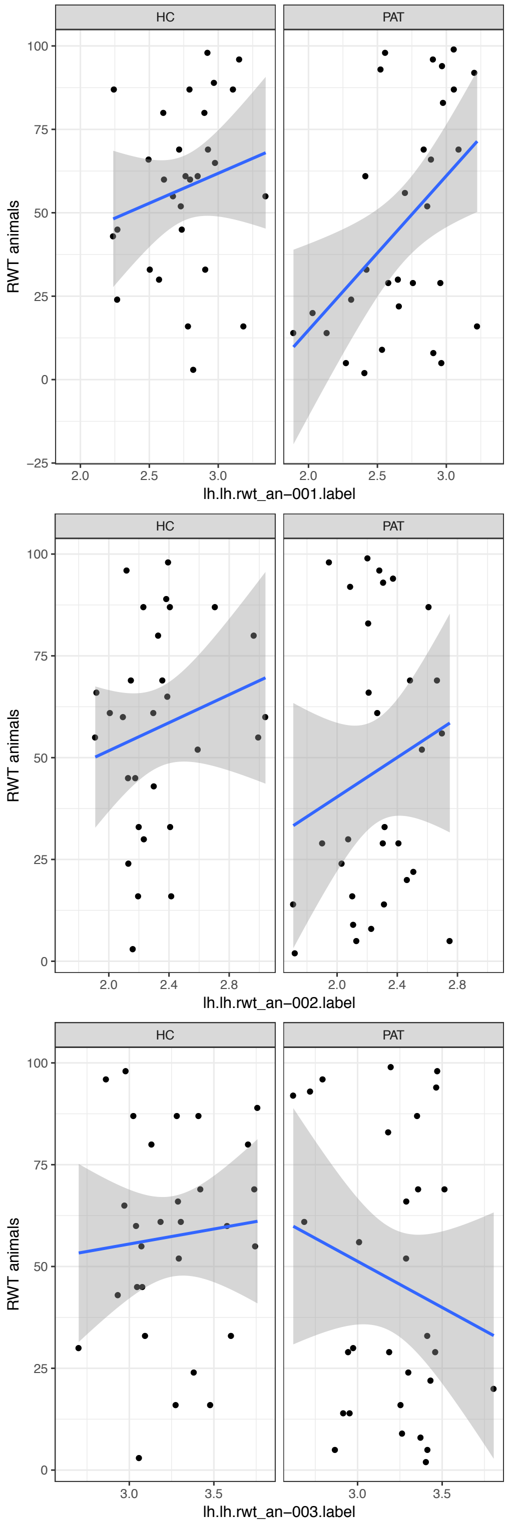

Dotplots with cortical thickness of each cluster and cognitive test performance, regression estimate (blue line) and its 95% confidence interval (grey area). Results for controls on the right, patients on the left

Figure A17 – Correlation between cortical thickness and VLMT verbal learning in clusters: controls and RRMS

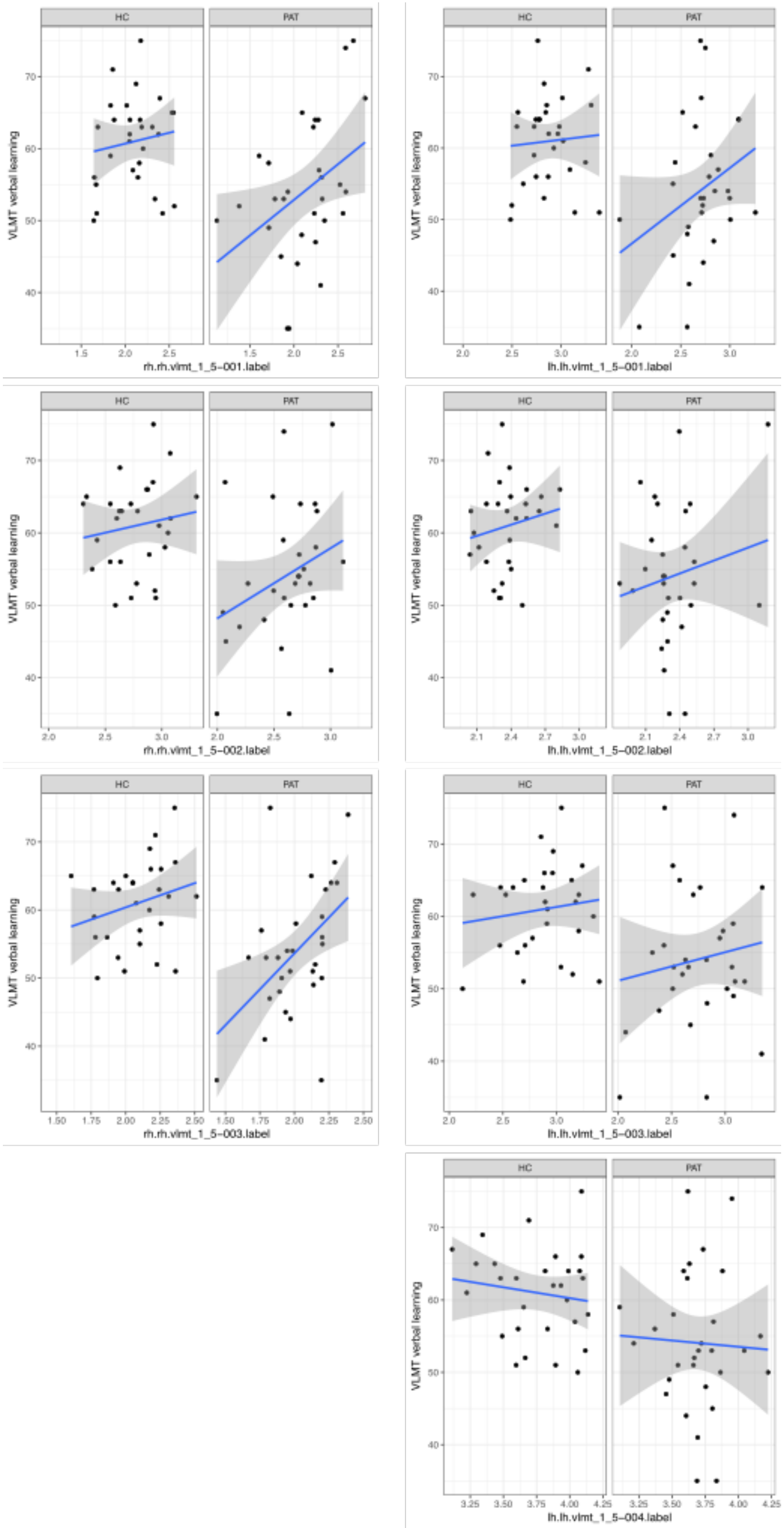

Dotplots with cortical thickness of each cluster and cognitive test performance, regression estimate (blue line) and its 95% confidence interval (grey area). Results for controls on the right, patients on the left
